# Music listening patterns and their associations with mood regulation, emotional processing, and cognitive performance: an intensive longitudinal questionnaire study

**DOI:** 10.3389/fpsyg.2026.1950103

**Published:** 2026-09-24

**Authors:** Yanyan Wang, Ge Cai, Tingting Wang

**Affiliations:** 1 Preschool Education College, Luoyang Normal University, Luoyang, Henan, China; 2 Student Affairs Office, Henan College of Forestry, Luoyang, China

**Keywords:** cognitive performance, experience sampling, individual differences, mood regulation, music listening

## Abstract

**Background:**

Music is often played when university students are studying and during their daily activities, but most of the research to date in the field of music listening and academic performance has used self-report techniques. Implications for student wellbeing and academic success are summarized where listening patterns are used to understand mood regulation and academic cognitive performance in real-world settings.

**Method:**

The design of this study was intensive longitudinal (IL) using experience sampling methodology (ESM) with event-contingent sampling over the 14-day period. Hundred and twenty-eight university students filled out questionnaires that measured musical sophistication, alexithymia, personality and trait mood regulation at baseline, and then answered short questions on 5,448 music listening episodes. Within-person and between-person associations in relation to academic versus non-academic contexts were examined using multilevel modeling.

**Results:**

Using music for mood regulation was the strongest predictor of mood improvement (*β* = 0.87, *p* < 0.001), followed by active enjoyment (*β* = 0.54, *p* < 0.001), while focus purposes predicted perceived academic cognitive performance (*β* = 0.56, *p* < 0.001). Musical sophistication moderated the mood regulation-mood change relationship (*β* = 0.22, *p* = 0.006), while alexithymia reduced its effectiveness (*β* = −0.19, *p* = 0.035). Lyrics presence was detrimental to cognitive performance during high-demand academic tasks (*β* = −0.28, *p* = 0.005), whereas faster tempos enhanced performance (*β* = 0.19, *p* = 0.035). Conscientiousness moderated the focus-cognitive performance relationship (*β* = 0.21, *p* = 0.018).

**Conclusion:**

Music listening patterns among university students are dynamically associated with mood regulation and academic cognitive performance in daily life, with effects moderated by individual differences including musical sophistication, alexithymia, and conscientiousness. These findings inform the development of personalized music-based interventions for student wellbeing and academic success.

## 1 Introduction

Music listening is a pervasive, goal-directed human behavior that engages auditory, affective, cognitive, and reward-related systems, making it a scientifically important context for studying mood regulation, emotional processing, and cognitive performance (Sharma et al., 2025; Qiao G. et al., 2026; Qiao S. et al., 2026; Reybrouck et al., 2021; Toader et al., 2023). Mood regulation refers to the use of music to modulate, sustain, or regulate affective states, whereas emotional processing encompasses the perception, appraisal, and neural encoding of emotionally salient musical stimuli. Cognitive performance includes functions such as attention, working memory, learning, and executive control. Musical sophistication is defined here in terms of self-reported musical abilities, training, engagement, emotional response to music, and perceptual abilities assessed using the Goldsmiths Musical Sophistication Index (Gold-MSI) (Huang et al., 2025). Alexithymia refers to difficulties in identifying, describing, and processing emotions and is assessed using the Toronto Alexithymia Scale (TAS-20).

Recent reviews indicate that listening is the dominant mode of music-based emotion regulation research, accounting for 85.1% of reviewed studies, and that the field has expanded rapidly since 2021, reflecting broad international interest in music as an everyday self-regulatory resource (Chong et al., 2024). This is a global phenomenon as music listening is not only cheap and scalable, but also embedded in culture and widely accepted during periods of stress, social disruptions, and academic demands, such as university students and community sample across multiple countries (Hennessy et al., 2021; Lee et al., 2026; Ribeiro et al., 2021).

Self-report, experience sampling, psychophysiology, and neuroimaging techniques have been used together in recent studies to measurably advance our understanding of the impact of music listening and music cognition. While previous research has demonstrated the engagement of distributed emotion-related networks during music processing, the temporal dynamics and functional interplay among these neural systems underlying effective responses to music remain incompletely understood (Trost et al., 2024). Neurophysiological evidence has also reported stronger and more consistent amygdala responses to live compared with recorded music, together with more widespread functional coupling across emotion-related systems during live music listening. These findings illustrate that emotional responses to music may depend on characteristics of the listening experience and its context.

Experimental and theoretical findings suggest that music influences cognition in a variety of ways: arousal processes may alter physiological and psychological activation, mood processes may influence positive mood states that can facilitate cognitive processing, and attentional processes may facilitate or interfere with limited cognitive resources depending on musical characteristics and task demands. These mechanisms are in line with arousal-mood and resource allocation theories (Giannouli et al., 2024). Although music listening has been linked to various positive effects in student populations at the behavioral level calm, happy music has been found to decrease induced negativity compared to sad music (Kachana et al., 2025; Liu et al., 2021). In parallel, clinical and non-clinical studies have been reviewed to show that music activities can influence mood, arousal, attention, memory, executive processes, and well-being, but this influence is dependent on the context, and the musical form (Dingle et al., 2021; Rajakumar and Mohan, 2024).

Notwithstanding this development, literature is conceptually and methodologically disunited. While music emotion regulation is often considered synonymous with music emotion research, there is still definitional ambiguity linking these terms, inconsistent operationalization of ‘emotion’, and music treated as a single type of exposure in contrast to the potential heterogeneity of music listening characteristics such as genre, valence, tempo, familiarity, context, intentionality or self-selection (Chong et al., 2024; Fan and Zhao, 2025; Wang et al., 2025). Diverse motives and strategies for listening have been identified in questionnaire surveys, such as diversion, solace, discharge, memory, calmness, and mood matching, but some of these constructs are not empirically distinct, making it difficult to determine which listening characteristics may be associated with mental health outcomes (Kahn et al., 2025; Morgan and Marroquín, 2024). Additional inconsistencies with regard to outcomes are also found: Overall music use to regulate emotions is not consistently related to depression or anxiety symptoms, however; mood-congruent music discharge is related to worse outcomes, and success in everyday affect regulation occurs in less than half of the listening episodes that were sampled and is strongly dependent on context and initial emotional state (Morgan and Marroquín, 2024; Randall et al., 2022). Furthermore, many studies utilize cross-sectional or retrospective self-report measures, which may lack representativeness and may not allow for causal inferences and ecological precision (Ahmed et al., 2026; Cai et al., 2026; Morgan and Marroquín, 2024).

There is a significant lack in the literature on the simultaneous investigation of music listening patterns, with regard to mood regulation, emotional processing and cognitive performance, in an ecologically valid framework. Past studies usually focus on a single outcome domain, are conducted in laboratory environments, employ retrospective recall techniques, and fail to consider individual difference moderators. To fill these gaps, the present study adopts an intensive longitudinal questionnaire design based on the Experience Sampling Methodology (ESM) over 14 days to measure the real-time music listening experience of university students. This methodological approach reduces reliance on retrospective recall, captures intra-individual variability, and allows examination of time-varying relationships. The novelty is the simultaneous investigation of several outcome domains in one analysis and testing of the role of the various factors of musical sophistication, alexithymia and personality traits as moderators.

The associations between personality traits and emotional regulation strategies and academic performance have been consistently found - particularly with the Big Five dimensions (Openness, Conscientiousness, Extraversion, Agreeableness, and Neuroticism) (Scarratt et al., 2026; Jiang, 2024). Of these factors, conscientiousness is noteworthy, as it is a personality trait that reflects self-discipline, organization and goal-setting behaviors that can have a significant impact on the use of music for improving academic concentration and achievement. On the other hand, neuroticism might affect the use of music for emotional regulation, as individuals with high levels of neuroticism may experience greater emotional instability and may differ in their use of music to regulate emotions (Stewart et al., 2019).

This study aims to create a more in-depth understanding of the dynamic process in which university students use music to shape their emotions and thinking process within their daily lives. To achieve this objective, we used an intensive longitudinal experience sampling design over a 14-day period to capture music listening experiences in real time. Specifically, this study seeks to: (1) explore intra-personal relationships between listening goals, music features and effects (mood change and perceived cognitive performance in the classroom); (2) determine whether effects are moderated by individual differences (musical sophistication, alexithymia, personality traits); and (3) examine whether effects vary depending on academic versus non-academic settings.

### 1.1 Rationale

In individuals with higher musical sophistication, music’s ability to enhance mood will be dampened as they are more emotionally sensitive to music, have better perceptual and analytical processing skills to evaluate musical structures, and will have more of a history with using music as an emotional medium. These attributes may facilitate the ability of music-savvy individuals to more accurately listen, interpret, and react to the emotional content of music, which can lead to increased emotional benefits from listening to music (Carvalho et al., 2022; Scarratt et al., 2026). On the other hand, alexithymia (defined as difficulties in emotional awareness and processing) can get in the way of the effective use of music to regulate mood. People with high alexithymia may not be able to identify how music impacts their emotional state or may be less likely to choose music that aligns with the way they need to regulate their emotions, leading to less effective music emotion regulation (Stewart et al., 2019; Morgan and Marroquín, 2024).

### 1.2 Hypothesis

The following directional hypotheses are proposed based on the theoretical framework of the arousal-mood hypothesis, resource allocation theory, and individual differences models.

*H1*: Music listening for mood regulation will be positively correlated with mood improvement, while background listening will have weaker or non-significant correlations with mood change. This forecast is based on evidence showing that music listening with a purpose and intention leads to more emotional benefits than passive or incidental listening (Randall et al., 2022; Stewart et al., 2019).

*H2*: Results indicated that music use for mood regulation and mood improvement would be positively moderated by musical sophistication, such that those with higher level of musical sophistication would demonstrate greater mood-enhancing effects from music listening. This forecast is based on studies that musical expertise improves emotional sensitivity and the capacity to experience emotional benefits from music (Carvalho et al., 2022; Scarratt et al., 2026).

*H3*: Music use for mood regulation will be negatively moderated by alexithymia scores, such that the more alexithymic (difficulty identifying and describing emotions) the less beneficial the mood regulation from music listening. This prediction is based on theoretical research indicating that dysfunction in emotional processing could hinder therapeutic outcomes of music (Morgan and Marroquín, 2024; Stewart et al., 2019).

*H4*: The relationship between music listening and perceived academic cognitive performance will be mediated by music characteristics (lyrics presence and tempo) and academic context. Specifically, we hypothesize that listening to music with words will have a negative effect on cognitive functioning during tasks of high cognitive demand (consistent with the resource allocation theory), and that faster music tempos will be beneficial for cognitive functioning during tasks of high cognitive demand (consistent with the arousal-mood hypothesis). Additionally, we hypothesize that focus-purpose listening will be more predictive of cognitive functioning in academic situations than in non-academic situations.

## 2 Literature review

The relevance of music as a model of emotional-cognitive interactions was confirmed by describing music listening as a multi-regional process in the brain that is relevant to emotional and cognitive functions (Toader et al., 2023). A review of 63 studies found that music activities, ranging from receptive listening to intentional listening, are correlated with various psychosocial processes, including arousal, mood, attention, memory, and social connection, thus creating a generalised framework for contemporary music-related well-being research (Dingle et al., 2021). Music listening has been the dominant theme in the emotion-regulation literature, and publications in the field jumped significantly from 2021 to 2024, reflecting both conceptual momentum and diversification of the methods used to study emotion regulation strategies (Chong et al., 2024). Music listening was situated in the broader emotion-regulation literature, and it was highlighted that the Music in Mood Regulation paradigm encompasses multiple different ways of regulating emotions, rather than just a single one (Morgan and Marroquín, 2024). This position was extended by showing that some purported emotion regulation strategies are only partially distinct empirically, while qualitative data identified additional regulatory functions such as remembering, calming oneself, and matching mood (Kahn et al., 2025).

Real-time fMRI demonstrated that live music elicited stronger and more consistent amygdala activation than recorded music and engaged a denser affective neural network, suggesting that ecological musical interaction can shape emotional processing more strongly than static stimuli (Trost et al., 2024). Calm and happy music more effectively reduced experimentally induced negative emotion than sad music, and further showed that negative mood interfered with conflict control performance on a Stroop task (Liu et al., 2021). Brief exposure to music affected visuospatial working memory, and it was argued that emotional activation and central executive attention are involved in shaping downstream cognitive performance, although the observed advantage was composer-specific rather than uniform across musical conditions (Giannouli et al., 2024).

Findings were nuanced by showing that in general there was no main effect of music on regulation intensity across strategies, but music decreased the perceived difficulty only in the reappraisal strategy, and effects were moderated by music sophistication and executive functioning (Carvalho et al., 2022). It was observed that 92.3% of surveyed students listened to music while studying and that many perceived benefits for concentration, relaxation, and study persistence, yet those findings remained descriptive and self-reported rather than performance-verified (Kachana et al., 2025).

Experience sampling found that affect-regulation goals were achieved in less than half of everyday listening episodes and that success was strongly shaped by context and baseline affect, challenging overly general claims that music listening is uniformly effective (Randall et al., 2022). Overall use of music as emotion regulation was not directly associated with depression and anxiety symptoms, whereas discharge-oriented listening showed positive associations with those symptoms through maladaptive regulation pathways (Morgan and Marroquín, 2024). Similarly, it was noted that distressed individuals tended to choose negatively valenced music that was not particularly effective for achieving well-being goals, even though music use overall was associated with positive affective change across countries (Hennessy et al., 2021). Several limitations across the field were highlighted, namely, the lack of a good theoretical separation between music emotion and music emotion regulation, the use of inconsistently defined terms, as well as the fact that only a few of the intra-musical elements that could explain the differential effects were drawn (Chong et al., 2024). Moreover, music preferences and listening contexts were associated with different psychological and behavioral patterns in students, thereby highlighting the importance of a multidimensional approach to listening behavior beyond simply counting the number of times students listen to music (Ahmed et al., 2026).

To extend the definition of music’s role in regulation beyond mood improvement, the qualitative findings of university students were integrated with their synthesized coping model, which included three additional values: emotional awareness, perceived control and interoceptive engagement (Lee et al., 2026). A large Brazilian cross-sectional sample showed that people with severe depressive symptoms more often used music for comfort, anxiety reduction, boredom relief, and cognitive stimulation, and their factor analysis distinguished negative mood management, positive mood management, cognitive functioning, and physical involvement as separable listening functions (Ribeiro et al., 2021).

Convergent evidence from systematic reviews indicated that music-based interventions can support attention, executive function, and episodic memory, but those findings derive largely from therapeutic or cognitively impaired populations and therefore cannot be directly generalized to healthy everyday listeners (Ito et al., 2022; Rajakumar and Mohan, 2024). It was further concluded that music-related benefits for executive functions are heterogeneous across interventions and cognitive domains, which strengthens the rationale for population-specific, ecologically grounded questionnaire studies of ordinary listening patterns (Rodriguez-Gomez and Talero-Gutiérrez, 2022). A cross-sectional framework was outlined in which frequency, duration, genre, and listening context can be linked to perceived focus, efficiency, retention, and qualitative motives, supporting the present study’s integrative design while leaving the empirical question open (Sharma et al., 2025).

Taken together, the recent literature supports the view that music listening is a multifaceted self-regulatory behavior with measurable associations across emotional and cognitive domains, but it also shows that outcomes depend on strategy, listener characteristics, context, and the specific listening pattern under study (Ahmed et al., 2026; Carvalho et al., 2022; Randall et al., 2022). The perspective of an active listener as an active regulator of internal states, as advocated by Reybrouck et al., is a more encompassing homeostatic one that is congruent with contemporary research on contextual and goal-dependent variability (Reybrouck et al., 2021). The importance of designs that reflect the natural contexts of auditory perception and separation of beneficial and maladaptive regulatory uses of media was emphasized, particularly in the face of interindividual differences in symptoms, goals, and demands of the context (Morgan and Marroquín, 2024; Randall et al., 2022). This study fills this gap by exploring music listening behaviors, as well as their relationships with mood regulation, emotional processing, and cognitive performance under a single framework, and expands previous research that has tended to study these fields in isolation or with more limited measures of music-related behaviors (Kachana et al., 2025; Kahn et al., 2025; Ribeiro et al., 2021).

## 3 Methodology

### 3.1 Study design

This study employed an intensive longitudinal design using Experience Sampling Methodology (ESM) with event-contingent sampling. Participants completed an initial baseline questionnaire followed by brief in-the-moment surveys triggered by music listening episodes over a 14-day period. This design is selected for its ability to capture the dynamic, context-dependent nature of everyday music listening, minimize retrospective recall bias, enable multilevel modelling to distinguish within-person from between-person effects, and provide ecological validity through data collection in natural environments. Event-contingent sampling ensures that data are collected specifically for music listening episodes rather than at random intervals, maximizing the relevance and efficiency of data collection. Data collection spanned 14 consecutive days, including both weekdays and weekends to capture variability in listening patterns across different academic and social contexts.

### 3.2 Participants

A target sample of 120–150 university students was planned for this study. With an expected completion of approximately 3–5 listening episodes per participant per day over 14 days, this yielded approximately 5,040–10,500 listening episodes for analysis, providing sufficient power for multilevel modelling. Participants must be currently enrolled university students aged 18 years or older, be regular music listeners who listen to music at least once per day, own a smartphone capable of receiving survey notifications, be willing to complete brief surveys during or immediately after music listening episodes, and be proficient in the language of the survey materials. Students currently receiving treatment for serious mental health conditions that may affect music listening experiences were excluded. Participants were recruited through university research participation programs, campus-wide email lists, student organization announcements, and social media platforms targeting university student populations.

### 3.3 Measures

#### 3.3.1 Baseline questionnaire

Testing for the 14-day ESM period occurred after the baseline questionnaire is completed, which assessed stable individual difference variables. Academic information (i.e., year of study, field of study, grade point average (GPA), typical amount of study hours/week) was gathered with custom items. Demographic data (such as age, gender and music listening history) also was gathered. Musical sophistication was measured using the Goldsmiths Musical Sophistication Index (Gold-MSI), a 39-item self-report inventory that assesses musical skills, training, engagement, emotions, and perceptual abilities. The Gold-MSI has demonstrated good internal consistency in previous research (Cronbach’s α = 0.85–0.92 for subscales) and correlates with objective measures of musical ability.

Emotional processing was assessed using the Toronto Alexithymia Scale (TAS-20), a 20-item measure of difficulty identifying and describing feelings, and externally oriented thinking. The TAS-20 has shown good psychometric properties across diverse samples, with established three-factor structure and internal consistency typically exceeding α = 0.80. Personality traits were measured using the Ten-Item Personality Inventory (TIPI), a brief measure of the Big Five personality dimensions. Despite its brevity, the TIPI has demonstrated adequate test–retest reliability and convergent validity with full-length personality measures in university student samples. Trait mood regulation using music was assessed with the Music in Mood Regulation (MMR) Scale, a 21-item measure of specific mood regulation strategies employed through music listening. The MMR scale has demonstrated satisfactory internal consistency across its subscales (α = 0.74–0.79) and has been validated in multiple cultural contexts. A comprehensive summary of all study measures and instruments is presented in Table 1.

**Table 1:** Summary of study measures and instruments.

**Construct**	**Measure**	**Description**	**Number of items**	**Response format**	**Administration**
Demographics and academics	Custom items	Age, gender, year of study, field of study, GPA, study hours	8	Multiple choice, open-ended	Baseline
Musical sophistication	Goldsmiths musical sophistication index (Gold-MSI)	Assesses musical skills, training, engagement, emotions, and perceptual abilities	39	7-point Likert scale	Baseline
Emotional processing	Toronto alexithymia scale (TAS-20)	Measures difficulty identifying and describing feelings, and externally oriented thinking	20	5-point Likert scale	Baseline
Personality	Ten-item personality inventory (TIPI)	Brief measure of Big Five personality traits	10	7-point Likert scale	Baseline
Trait mood regulation	Music in mood regulation scale (MMR)	Measures specific mood regulation strategies using music	21	5-point Likert scale	Baseline
Contextual information	Custom ESM items	Location, primary academic activity, companionship status	4	Categorical	ESM (each episode)
Academic context	Custom ESM item	Whether listening occurred during academic vs. non-academic activity	1	Categorical	ESM (each episode)
Music characteristics	Custom ESM items	Genre, familiarity, enjoyment, lyrics presence, tempo, source	6	Categorical, 7-point Likert	ESM (each episode)
Listening purpose	Custom ESM item	Purpose of listening (mood regulation, background, active enjoyment, focus, social, other)	1	Multiple select	ESM (each episode)
Intentionality	Custom ESM item	Deliberateness of music choice	1	7-point Likert	ESM (each episode)
Pre-listening mood	Custom ESM items	Valence and Arousal before listening	2	7-point Likert	ESM (each episode)
Post-listening mood	Custom ESM items	Valence and Arousal after listening	2	7-point Likert	ESM (each episode)
Perceived academic cognitive performance	Custom ESM items	Perceived focus, task performance, cognitive demand during academic tasks	3	7-point Likert	ESM (each episode)
Emotional regulation	Custom ESM items	Mood regulation goal, regulation success	2	Categorical, 7-point Likert	ESM (each episode)

#### 3.3.2 Experience sampling questionnaire

The experience sampling questionnaire was administered each time a participant reports a music listening episode. Items are brief to minimize participant burden while capturing key variables. Contextual information included the time of the listening episode, location with categories including home, campus or library, commuting, exercise, social setting, and other, primary academic-related activity with categories including studying, attending lectures, completing assignments, commuting, exercising, relaxing, socializing, and other, and companionship status with categories including alone, with friends, with classmates, with family, with partner, in public, and other. An additional item assessed whether music listening occurred during academic-related activities versus non-academic activities.

Music characteristics were assessed through items on genre selected from a list of common music genres, familiarity rated on a scale from 1 (not at all familiar) to 7 (very familiar), enjoyment rated on a scale from 1 (not at all) to 7 (very much), presence of lyrics with response options of yes, no, or instrumental, tempo rated on a scale from 1 (very slow) to 7 (very fast), and source of music selection with categories including playlist created by the participant, algorithm recommendation, friend or other person, radio, and other.

Listening purpose and intentionality were assessed by asking participants why they chose to listen to music, with the option to select all that applied from mood regulation, background, active enjoyment, focus or concentration (including studying), social or cultural, and other. Intentionality was rated on a scale from 1 (not at all deliberate) to 7 (very deliberate). Mood assessment was conducted both before and after listening to capture mood changes. Pre-listening mood was assessed for valence with the question “Before listening, how did you feel?” rated on a scale from 1 (not at all positive) to 7 (very positive) and for arousal with the question “Before listening, how did you feel?” rated on a scale from 1 (not at all energetic) to 7 (very energetic). Post-listening mood was assessed similarly with items asking “Right now, how do you feel?” for both positivity and energy levels.

Perceived academic cognitive performance was assessed through items on perceived focus during academic tasks with the question “During this listening episode, how focused were you on your academic task?” rated on a scale from 1 (very distracted) to 7 (very focused), perceived task performance with the question “How well were you performing your academic task while listening?” rated on a scale from 1 (very poorly) to 7 (very well), and cognitive demand of academic activities with the question “How cognitively demanding was your primary academic activity?” rated on a scale from 1 (not at all demanding) to 7 (extremely demanding). Emotional regulation was assessed through items on mood regulation goal asking whether participants intended to change their mood through music with response options of yes, no, or unsure, and mood regulation success rated on a scale from 1 (not at all) to 7 (completely) in response to the question “To what extent did music help you achieve your mood goal?”

### 3.4 Procedure

#### 3.4.1 Pre-study phase

Potential participants completed a brief screening survey to assess eligibility based on the inclusion and exclusion criteria including current university student status. Eligible participants were provided with detailed information about the study and asked to provide informed consent electronically. Following consent, participants completed the baseline questionnaire online, which took approximately 20–30 min. After completion of the baseline questionnaire, participants received training and orientation on the ESM procedure, covering how to access the online survey platform, when to complete listening episode surveys, best practices for timely completion, and common troubleshooting issues. Finally, participants were shown how to set up the system, putting the selected ESM platform on their phones or saving the link to the web-based survey for quick access.

The complete list of ESM survey items and response options was enlisted in Supplementary Table S1, and the survey flow diagram was shown in Supplementary Figure S1. All ESM surveys were conducted on the Qualtrics survey platform (Qualtrics, Provo, UT) via a mobile-friendly web link participants saved to their mobile phone to complete as many daily as possible at the same time (morning and evening).

#### 3.4.2 Experience sampling phase

The experience sampling phase lasted for 14 days and used event-contingent sampling. After starting a music listening session, the participants were directed to the survey on the designated platform to fill out the first part of the survey, which included measures for initial mood, contextual and expectancy measures. Following the listening episode, participants filled out a short post-listening questionnaire, which measured post-listening mood, listening goals, music characteristics, and perceived academic cognitive outcomes. The first survey section measured initial mood, context and expectations. Participants filled out a short follow-up questionnaire after the listening episode to evaluate their mood after listening, the goal of the listening activity, the characteristics of the music, and their perceived academic cognitive outcomes after listening. The survey was designed for completion within 5 min of the listening episode. Participants were encouraged to complete surveys for all music listening episodes throughout the 14-day period. Regular reminder notifications were sent to participants, but these were not excessive to avoid burden. Participants received clear instructions and training, incentives for consistent participation such as course credit or monetary compensation, and prompt support for technical issues.

#### 3.4.3 Post-study phase

Following the 14-day ESM period, participants completed a short debrief questionnaire asking about their experience with the ESM procedure, including items assessing whether the surveys influenced their listening habits as a reactivity check. Participants received compensation proportional to their completion rate, either course credit or monetary compensation depending on the recruitment channel.

### 3.5 Data analysis

#### 3.5.1 Compliance criteria

*A priori*, a compliance rate of 20% of the expected number of surveys (based on the number of days participants were active in the study and an expected number of surveys of 3–5 per day) was set for inclusion in the final analytical sample, which was based on the criterion that compliance rates in previous ESM studies usually range between 10 and 20% of expected surveys, and thus yield reliable multilevel estimates (Janssens and Kraft, 2012).

#### 3.5.2 Missing data handling

To deal with missing data, multiple imputation with chained equations (MICE) was used for baseline variables where less than 5% of data were missing. For ESM variables, missing data were addressed at the level of the multilevel models by full information maximum likelihood estimation (FIML), which provided an estimation of the model parameters using all available data without needing to delete individual observations. To assess the robustness of the results to handling of missing data, the models were compared with and without imputation, via sensitivity analyses.

#### 3.5.3 Preliminary and multilevel analyses

The data had a hierarchical structure with listening episodes at Level 1 nested within participants at Level 2. Preliminary analyses included descriptive statistics and scale reliability calculated using Cronbach’s alpha for multi-item measures at baseline. Intraclass correlation coefficients were calculated to quantify the variance at Level 1 (within person) compared to the variance at Level 2 (between persons). Data were analyzed using a multilevel or mixed effects model, which allowed for the partitioning of within-person from between-person effects and took account of the nested nature of the data. Within-person changes in mood and perceived academic cognitive performance were the primary focus of analysis, with listening purpose, music characteristics, and context as predictors. All continuous Level 1 predictors were person-mean centered to separate within-person from between-person effects. Cross-level interactions were tested to examine whether individual differences including musical sophistication, alexithymia, personality, and academic year moderated the within-person associations between listening patterns and outcomes.

#### 3.5.4 Contextual and temporal analyses

Contextual effects were examined to determine whether associations differed by academic versus non-academic activities. Temporal dynamics were modeled to examine time trends in listening patterns and outcomes across the 14-day period, with particular attention to patterns during examination or high-stakes academic periods.

#### 3.5.5 Model estimation and secondary analyses

Models were estimated using Restricted Maximum Likelihood or Maximum Likelihood estimation in statistical software such as R using the lme4 package or SPSS using the MIXED or GENLINMIXED procedures. Secondary analyses included exploratory factor analysis to examine the factor structure of listening purposes and music characteristics, mediation analysis to test whether mood change mediated the relationship between listening purpose and perceived academic cognitive performance, and growth curve modeling to examine whether the effect of music listening on outcomes changed over the course of the study period.

### 3.6 Statistical analyses

The multilevel models testing our hypotheses are the key analyses, but supplementary exploratory analyses were also carried out to gain more insights into the data structure. In particular, exploratory factor analysis was performed to validate our measurement approach by analyzing the factor structure of listening purposes and music characteristics. As exploratory follow up analyses, mediation analyses were performed to assess whether the relationship between listening purpose and perceived academic cognitive performance is mediated by mood change. Such analyses were not focus of the hypothesis testing, but were complementary to the primary analyses in order to gain additional insights into the mechanisms underlying the observed effects. All exploratory analyses are separated from primary hypothesis-testing analyses in the result section.

## 4 Results

### 4.1 Participant characteristics

A total of 128 university students completed the baseline questionnaire and commenced the 14-day experience sampling period. The final analytical sample consisted of 124 participants who completed at least 20% of expected ESM episodes, yielding 5,024 listening episodes for analysis. The CONSORT flow diagram (Figure 1) depicts participant recruitment, enrolment and completion across the study. Participant ages ranged from 18 to 35 years with a mean age of 21.4 years (SD = 3.2). The sample was approximately balanced by gender, with 58 males (45.3%), 65 females (50.8%), and 5 individuals identifying as non-binary or other (3.9%). Regarding year of study, 25.0% were first-year students, 21.9% were second-year students, 18.8% were third-year students, 17.2% were fourth-year students, and 17.2% were graduate or postgraduate students. In terms of field of study, 32.8% were enrolled in humanities or social sciences, 29.7% in STEM fields, 21.9% in business or economics, 9.4% in arts or music, and 6.3% in other fields. The mean grade point average was 3.2 (SD = 0.5) out of 4.0, and participants reported studying an average of 18.4 h per week (SD = 8.7). The baseline characteristics are presented in Table 2.

**Figure 1:**
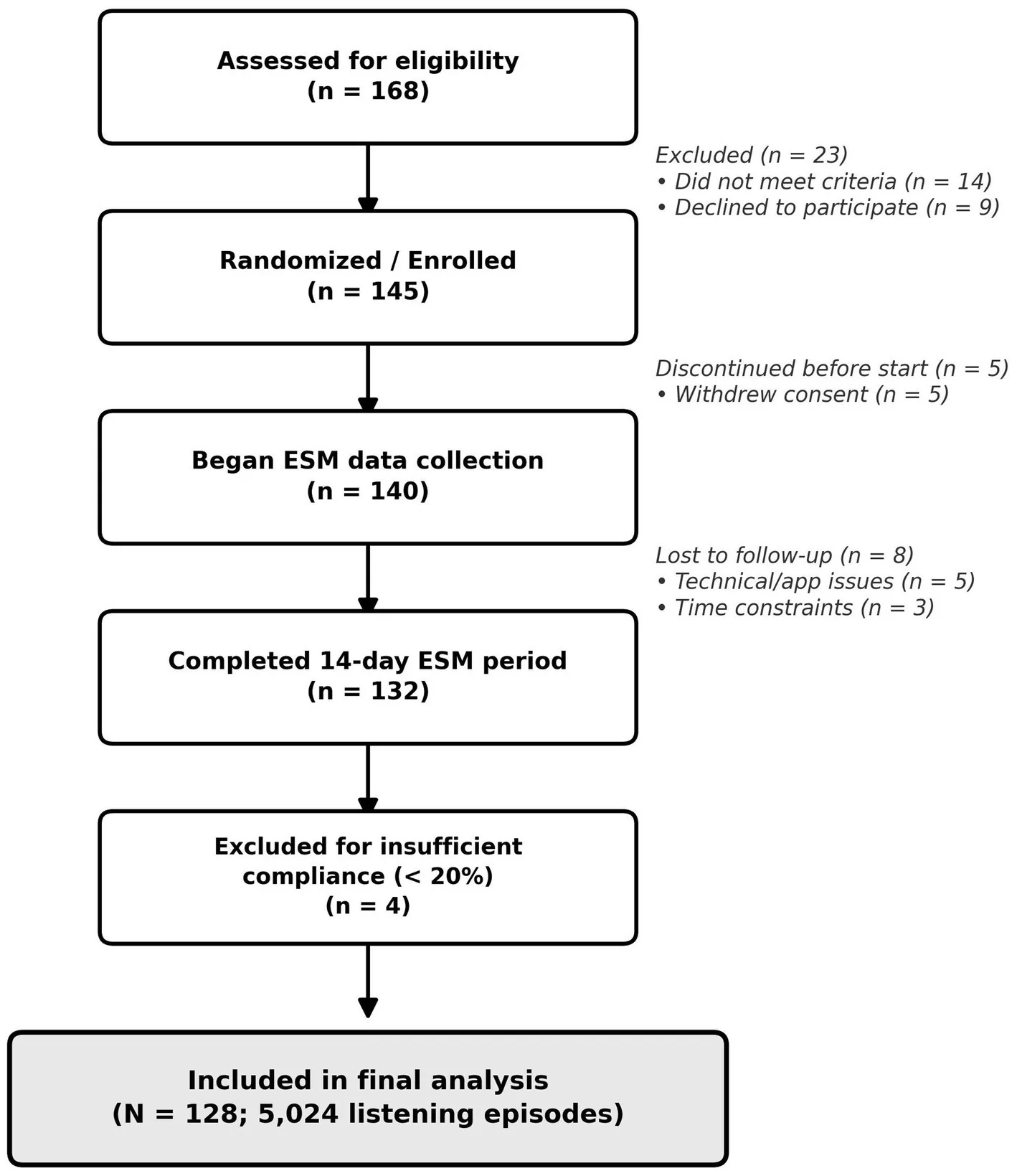
CONSORT flow chart. Flow diagram illustrating participant recruitment, enrollment, allocation, and completion through the study. Numbers represent participants at each stage of the study. Reasons for exclusion and dropout are specified.

**Table 2:** Participant demographics and baseline characteristics.

Characteristic	Category	*N*	%	Mean (SD)	Range
Age	–	128	-	21.4 (3.2)	18–35
Gender	Male	58	45.3	–	–
	Female	65	50.8	–	–
	Non-binary/Other	5	3.9	–	–
Year of study	First year	32	25.0	–	–
	Second year	28	21.9	–	–
	Third year	24	18.8	–	–
	Fourth year	22	17.2	–	–
	Graduate/Postgraduate	22	17.2	–	–
Field of Study	Humanities/Social sciences	42	32.8	–	–
	STEM	38	29.7	–	–
	Business/Economics	28	21.9	–	–
	Arts/Music	12	9.4	–	–
	Other	8	6.3	–	–
GPA (out of 4.0)	–	–	–	3.2 (0.5)	2.0–4.0
Study hours per week	–	–	–	18.4 (8.7)	5–45
Daily music listening duration (hours)	–	–	–	3.2 (2.1)	0.5–10.0
Musical Training (years)	–	–	–	4.2 (5.1)	0–25
Gold-MSI total score	–	–	–	81.8 (18.6)	39–126
TAS-20 total score	–	–	–	49.2 (11.5)	20–78
TIPI openness	–	–	–	5.3 (1.2)	1–7
TIPI conscientiousness	–	–	–	4.6 (1.5)	1–7
TIPI extraversion	–	–	–	4.4 (1.6)	1–7
TIPI agreeableness	–	–	–	5.0 (1.3)	1–7
TIPI neuroticism	–	–	–	4.6 (1.5)	1–7
MMR entertainment	–	–	–	3.9 (0.9)	1–5
MMR diversion	–	–	–	3.6 (1.0)	1–5
MMR discharge	–	–	–	3.2 (1.1)	1–5
MMR solace	–	–	–	3.8 (0.9)	1–5

Gold-MSI = Goldsmiths Musical Sophistication Index; TAS-20 = Toronto Alexithymia Scale; TIPI = Ten-Item Personality Inventory; MMR = Music in Mood Regulation Scale.

Participants reported a mean daily music listening duration of 3.2 h (SD = 2.1) and a mean of 4.2 years (SD = 5.1) of formal musical training. The Goldsmiths Musical Sophistication Index total score averaged 81.8 (SD = 18.6) out of a possible 126, indicating moderate to high levels of musical sophistication in the student sample. The mean score for the Toronto Alexithymia Scale was 49.2 (SD = 11.5) indicating relatively low alexithymia scores. Overall, the personality trait scores were not extreme or high, with openness (*M* = 5.3, SD = 1.2) and agreeableness (*M* = 5.0, SD = 1.3) having the highest mean scores. The most common mood regulation strategies with music measured with the Music in Mood Regulation Scale were using music for solace (*M* = 3.8, SD = 0.9) and entertainment (*M* = 3.9, SD = 0.9). Figure 2 provides the conceptual framework for the study.

**Figure 2:**
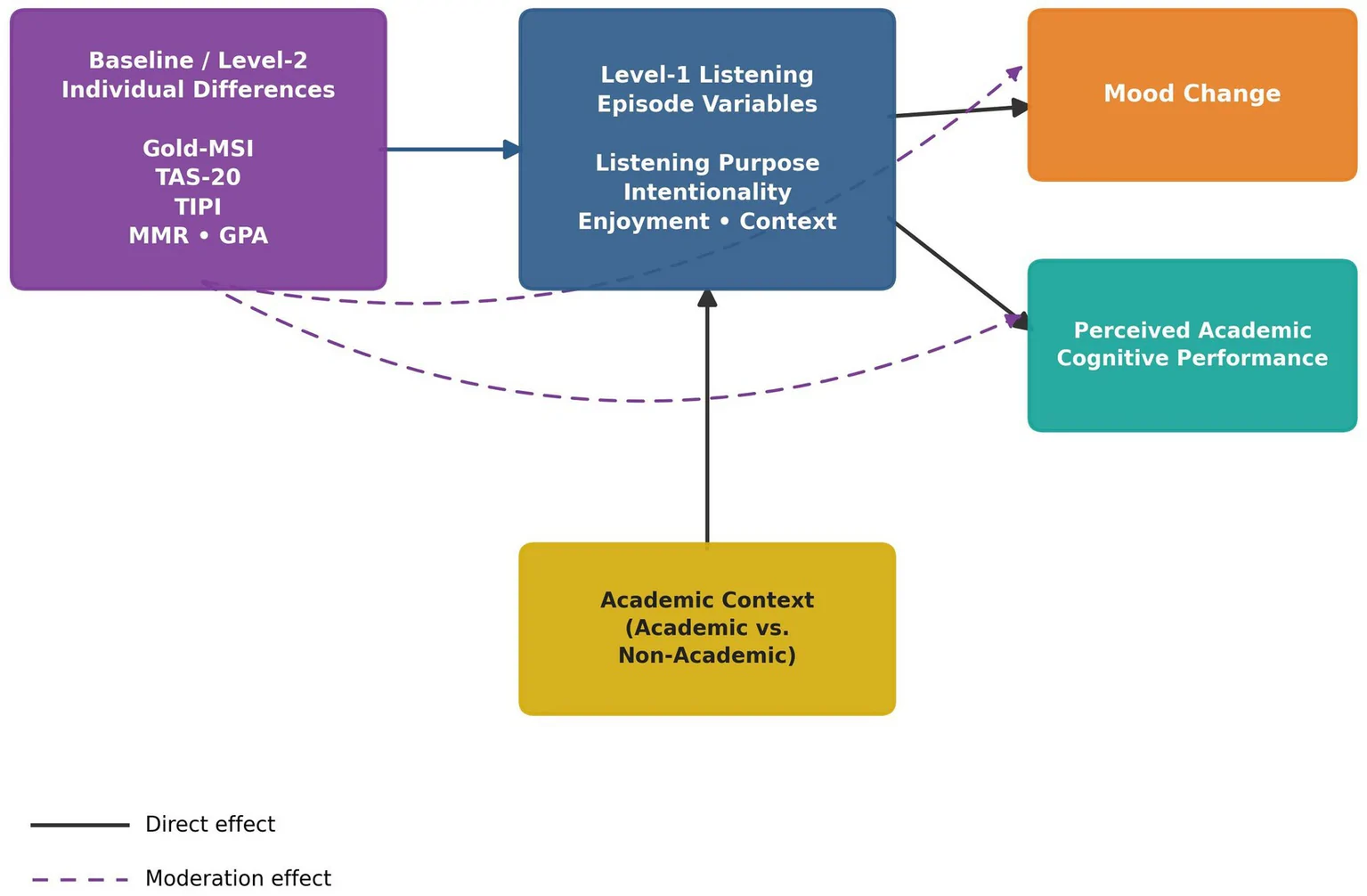
Conceptual framework. Conceptual framework depicting the hypothesized relationships among study variables. Solid arrows represent direct effects, dashed arrows represent moderation effects. Gold-MSI = Goldsmiths Musical Sophistication Index; TAS-20 = Toronto Alexithymia Scale; TIPI = Ten-Item Personality Inventory; MMR = Music in Mood Regulation Scale; GPA = Grade Point Average.

### 4.2 Descriptive statistics of ESM variables

Descriptive statistics and intraclass correlation coefficients for all variables from the experience sampling method for the 14-day study period are reported in Table 3. The mean number of listening episodes participants completed per day was 4.3 (SD = 1.9 between-person; SD = 1.6 within-person) and the mean length of the listening episodes was 27.8 min per episode (SD = 13.8 between-person; SD = 11.5 within-person). Participants reported moderate to high familiarity of musical characteristics (*M* = 5.3, SD = 1.3), high enjoyment (*M* = 5.7, SD = 1.1) and moderate tempo (*M* = 4.5, SD = 1.4). On the intentionality of music choice scale, the mean score was 4.1 (SD = 1.6) which represents moderate deliberation in music selection. As seen in Figure 3, listening episodes were evenly distributed across the 14 days of the study with more on the weekdays than the weekends of the study.

**Table 3:** Descriptive statistics of ESM variables.

Variable	Level	Mean (SD)	ICC	Min	Max
Listening episodes per day	Between-person	4.3 (1.9)	0.29	1.0	8.8
	Within-person	4.3 (1.6)	–	0	12.0
Listening duration (Minutes)	Between-person	27.8 (13.8)	0.34	5.0	75.0
	Within-person	27.8 (11.5)	–	2.0	120.0
Familiarity	Between-person	5.3 (1.3)	0.41	2.0	7.0
	Within-person	5.3 (1.2)	–	1.0	7.0
Enjoyment	Between-person	5.7 (1.1)	0.37	2.5	7.0
	Within-person	5.7 (0.9)	–	1.0	7.0
Tempo	Between-person	4.5 (1.4)	0.30	1.5	6.8
	Within-person	4.5 (1.3)	-	1.0	7.0
Intentionality	Between-person	4.1 (1.6)	0.28	1.2	6.9
	Within-person	4.1 (1.4)	–	1.0	7.0
Pre-listening mood (valence)	Between-person	4.2 (1.3)	0.32	1.8	6.5
	Within-person	4.2 (1.2)	–	1.0	7.0
Pre-listening mood (arousal)	Between-person	4.0 (1.4)	0.29	1.5	6.8
	Within-person	4.0 (1.3)	–	1.0	7.0
Post-listening mood (valence)	Between-person	5.1 (1.2)	0.35	2.2	6.9
	Within-person	5.1 (1.1)	–	1.0	7.0
Post-listening mood (arousal)	Between-person	4.7 (1.3)	0.31	2.0	7.0
	Within-person	4.7 (1.2)	-	1.0	7.0
Perceived academic focus	Between-person	4.3 (1.5)	0.26	1.5	6.8
	Within-person	4.3 (1.4)	–	1.0	7.0
Academic task performance	Between-person	4.5 (1.4)	0.29	1.8	6.9
	Within-person	4.5 (1.3)	–	1.0	7.0
Academic cognitive demand	Between-person	4.6 (1.5)	0.35	1.2	6.9
	Within-person	4.6 (1.3)	–	1.0	7.0
Mood regulation success	Between-person	4.5 (1.5)	0.36	1.5	6.8
	Within-person	4.5 (1.3)	–	1.0	7.0

ICC = Intraclass Correlation Coefficient. ICC values represent the proportion of variance attributable to between-person differences.

**Figure 3:**
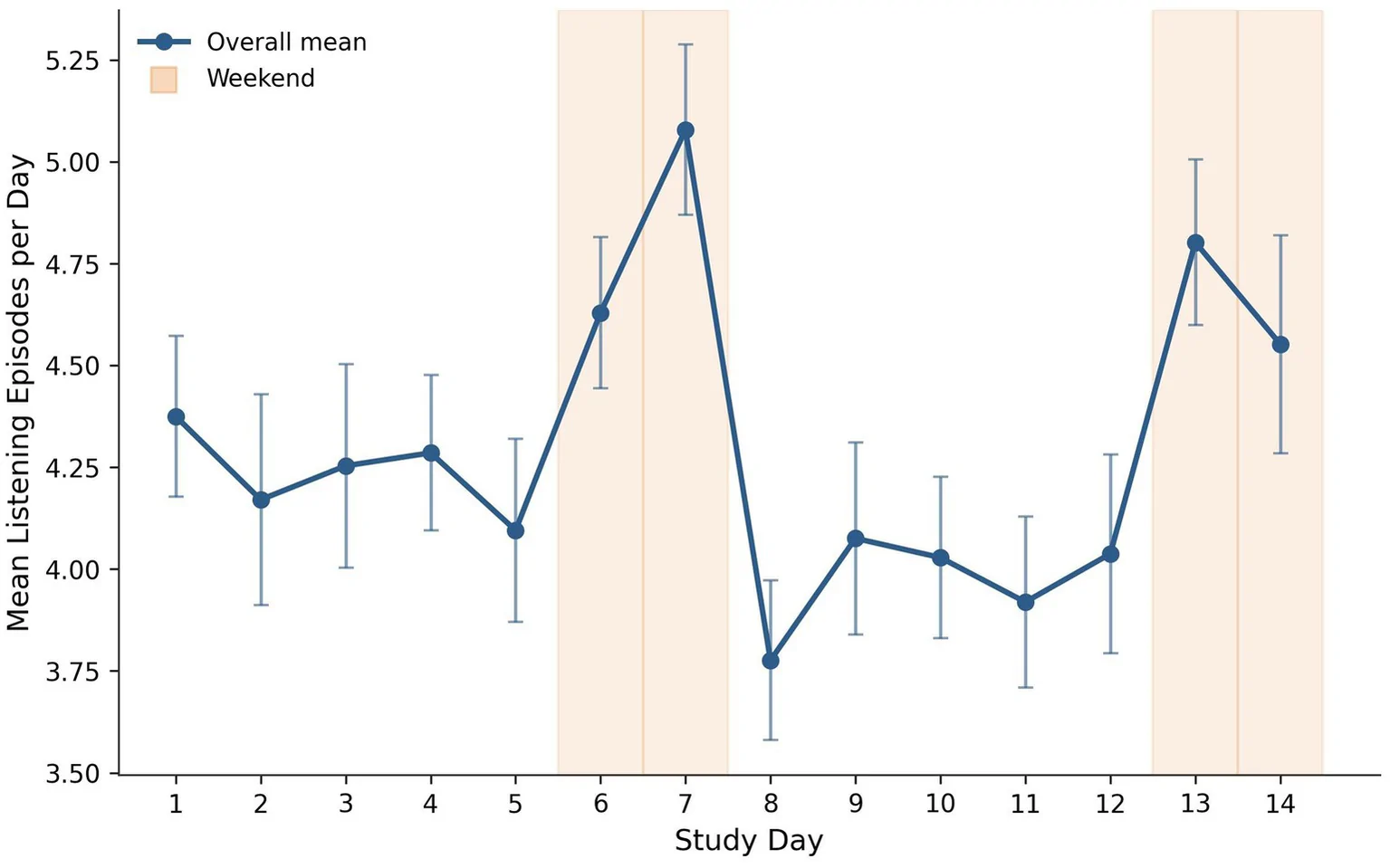
Distribution of listening episodes across the study period. Mean number of listening episodes per participant per day across the 14-day study period. Separate lines represent weekdays and weekends. Error bars represent standard errors. The figure illustrates participant compliance and temporal trends in listening frequency among university students.

Before listening, the mood valence was moderately positive (*M* = 4.2, SD = 1.3) and the mood arousal was moderately positive (*M* = 4.0, SD = 1.4). After music listening, valence (*M* = 5.1, SD = 1.2) and arousal (*M* = 4.7, SD = 1.3) both significantly increased compared to pre-listening levels [valence: *t*(123) = 12.45, *p* < 0.001; arousal: *t*(123) = 8.92, *p* < 0.001]. The mean scores of perceived academic cognitive performance variables were in the moderate to high range; perceived academic focus (4.3, SD = 1.5), academic task performance (4.5, SD = 1.4), and academic cognitive demand (4.6, SD = 1.5). Mood regulation success averaged 4.5 (SD = 1.5), suggesting moderate perceived effectiveness of music for mood regulation among university students.

Intraclass correlation coefficients (ICCs) represent the proportion of total variance attributable to between-person differences. To express this as a percentage of variance, the ICC value must be squared (ICC^2^ × 100). For example, an ICC of 0.41 indicates that 16.8% (0.41^2^ × 100) of the total variance is attributable to between-person differences, while the remaining 83.2% is attributable to within-person fluctuations. In the present study, ICCs showed that enjoyment (ICC = 0.37) and familiarity (ICC = 0.41) were relatively more trait-like, whereas perceived academic focus (ICC = 0.26) and intentionality (ICC = 0.28) exhibited greater state-like fluctuations, with between-person variance accounting for only 6.8–16.8% of total variance and the remainder due to within-person changes. This substantial within-person variability justified the use of multilevel modeling to separately examine within-person and between-person effects on outcomes such as mood and perceived academic cognitive performance. The ICC values thus confirmed that meaningful variation in outcomes was statistically explained by fluctuations in listening patterns and contexts within individuals over time.

### 4.3 Contextual patterns of music listening

The frequency and distribution of listening contexts in the 5,024 listening episodes is shown in Table 4. Music was listened to most in the home (52.0%), followed by campus or library (23.0%), commuting (12.0%), exercise (8.0%), social (3.0%) and other (2.0%). Primary activities included studying (27.0%), relaxing (20.0%), commuting (12.0%), attending lectures (9.0%), doing assignments (8.0%), exercising (8.0%), socializing (8.0%) and other activities (8.0%). Remarkably, 44.0% of the listening episodes took place during Academic activities and 56.0% during Non-Academic activities. Listening episodes occurred with no one else present (64.0%) and with companions including: friends (15.0%), classmates (8.0%), family (7.0%), partner (4.0%) and in the public (2.0%). The stacked bar chart (Figure 4A) shows that the most common listening setting for most activities was the home environment but the setting for the commuting and exercise activities was the other way around.

**Table 4:** Frequency and distribution of listening contexts.

Context variable	Category	Frequency (*N*)	Percentage (%)
Location	Home	2,612	52.0
	Campus/Library	1,156	23.0
	Commuting	603	12.0
	Exercise	402	8.0
	Social setting	151	3.0
	Other	100	2.0
Primary activity	Studying	1,356	27.0
	Attending lectures	452	9.0
	Completing assignments	402	8.0
	Commuting	603	12.0
	Exercising	402	8.0
	Relaxing	1,005	20.0
	Socializing	402	8.0
	Other	402	8.0
Academic vs. Non-academic	Academic activity	2,210	44.0
	Non-academic activity	2,814	56.0
Companionship	Alone	3,216	64.0
	With friends	754	15.0
	With classmates	402	8.0
	With family	352	7.0
	With partner	201	4.0
	In public	100	2.0

Total listening episodes = 5,024 across 128 participants.

**Figure 4:**
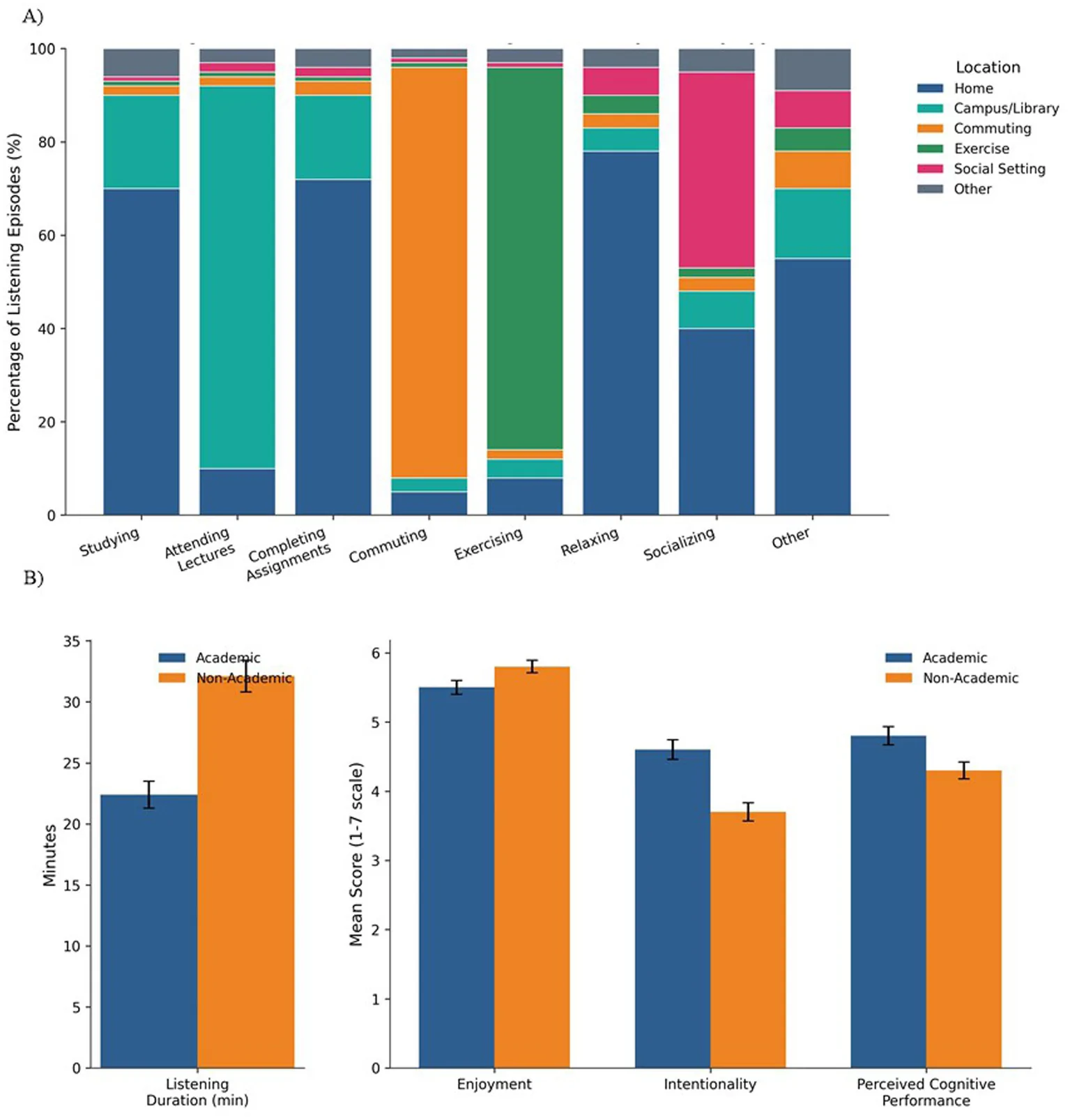
Distribution of listening contexts by activity type. Stacked bar chart showing the distribution of listening contexts across activity types. **(A)** The *x*-axis represents primary activity categories, and the *y*-axis represents the percentage of listening episodes. Different colors represent different locations. The figure reveals patterns in where and when music listening occurs in university students’ daily lives, with particular attention to academic versus non-academic contexts. **(B)** Paired bar chart comparing music listening patterns during academic versus non-academic activities. The *y*-axis represents mean scores for listening duration, enjoyment, intentionality, and perceived cognitive performance. Error bars represent standard errors. Significant differences were observed for listening duration, intentionality, and perceived cognitive performance between academic and non-academic contexts among university students.

The frequency and distribution of music characteristics according to listening episodes are given in Table 5. The most frequently reported genres were pop (25.0%), rock (19.0%), electronic or dance (13.0%), classical (10.0%), hip-hop or rap (9.0%), folk or acoustic (7.0%), R&B or soul (6.0%), jazz (4.0%), metal (3.0%), and other genres (4.0%). Lyrics were more likely than instrumental music or mixed content to appear in an episode (71.0% of episodes). As far as the sources of music selection, the most popular source was personal playlists (45.0%), followed by algorithm recommendations (25.0%), radio (10.0%), friends or other people (9.0%) and other sources (11.0%).

**Table 5:** Frequency and distribution of music characteristics.

Music characteristic	Category	Frequency (*N*)	Percentage (%)
Genre	Pop	1,256	25.0
	Rock	954	19.0
	Classical	502	10.0
	Electronic/Dance	653	13.0
	Hip-Hop/Rap	452	9.0
	Jazz	201	4.0
	Folk/Acoustic	352	7.0
	Metal	150	3.0
	R&B/Soul	302	6.0
	Other	202	4.0
Lyrics Presence	Yes	3,568	71.0
	No (Instrumental)	1,055	21.0
	Mixed	401	8.0
Source	My Playlist	2,261	45.0
	Algorithm Recommendation	1,256	25.0
	Friend/Other person	452	9.0
	Radio	502	10.0
	Other	553	11.0

Total listening episodes = 5,024 across 128 participants.

As shown in Table 6, the listening purpose frequency and distribution are shown. Percentages may add up to more than 100% because participants were eligible to choose more than one purpose per episode. The most common reason given was mood regulation (48.0%) followed by background listening (43.0%), active enjoyment (29.0%), focus or concentration studying (21.0%), social or cultural reasons (6.0%), and other reasons (4.0%). The results of the distribution show that the main motives for music listening among university students were mood regulation and background listening.

**Table 6:** Frequency and distribution of listening purposes.

Listening purpose	Frequency (*N*)	Percentage (%)
Mood regulation	2,412	48.0
Background listening	2,160	43.0
Active enjoyment	1,457	29.0
Focus/concentration (including studying)	1,055	21.0
Social/cultural	302	6.0
Other	201	4.0

Total listening episodes = 5,024. Participants could select multiple purposes per listening episode, therefore percentages sum to greater than 100%.

### 4.4 Academic versus non-academic listening patterns

The paired bar chart in Figure 4B displays the difference in music listening habits between schoolwork and non-schoolwork. Many differences were found between the variables. Listening duration was longer during non-academic activities (*M* = 31.2 min, SD = 12.8) compared to academic activities (*M* = 24.6 min, SD = 10.4; *t* = 4.82, *p* < 0.001). Intentionality was higher during academic activities (*M* = 4.5, SD = 1.5) compared to non-academic activities (*M* = 3.8, SD = 1.6; *t* = 3.96, *p* < 0.001). Enjoyment was slightly higher during non-academic activities (*M* = 5.8, SD = 1.0) compared to academic activities (*M* = 5.5, SD = 1.1; *t* = 2.34, *p* = 0.019). The perceived cognitive performance was found to be significantly lower during academic activities (*M* = 4.1, SD = 1.3) than during non-academic activities (*M* = 4.8, SD = 1.2; *t* = 5.12, *p* < 0.001). The results showed that the listening habits of university students were adjusted according to academic requirement of the activity, longer listening duration when doing academic tasks with higher cognitive performance compared to other activities, and shorter listening duration and lower cognitive performance when listening to music during academic tasks.

### 4.5 Bivariate correlations

Table 7 shows the bivariate correlations among baseline individual difference and aggregated ESM variables. The Gold-MSI was significantly positively associated with mood change (*r* = 0.32, *p* < 0.001), perceived academic focus (*r* = 0.22, *p* < 0.05), academic task performance (*r* = 0.26, *p* < 0.01), enjoyment (*r* = 0.28, *p* < 0.01), and mood regulation success (*r* = 0.30, *p* < 0.01). Conversely, alexithymia as measured by the TAS-20 showed significant negative correlations with mood change (*r* = −0.27, *p* < 0.01), perceived academic focus (*r* = −0.18, *p* < 0.05), academic task performance (*r* = −0.20, *p* < 0.05), enjoyment (*r* = −0.22, *p* < 0.05), and mood regulation success (*r* = −0.25, *p* < 0.01). The music listening benefits were higher for higher musical sophistication and lower alexithymia for university students.

**Table 7:** Bivariate correlations between baseline and ESM variables.

Variable	1	2	3	4	5	6	7	8	9	10	11	12	13	14
1. Gold-MSI	–													
2. TAS-20	−0.40***	–												
3. TIPI-O	0.36***	−0.28**	–											
4. TIPI-C	0.16	−0.20*	0.14	–										
5. TIPI-E	0.24**	−0.30**	0.34***	0.18*	–									
6. TIPI-A	0.15	−0.22*	0.26**	0.18*	0.20*	–								
7. TIPI-N	−0.20*	0.40***	−0.17	−0.24**	−0.36***	−0.28**	–							
8. MMR-total	0.42***	−0.34***	0.30**	0.11	0.28**	0.18*	−0.26**	–						
9. Mood change	0.32***	−0.27**	0.20*	0.13	0.17*	0.10	−0.18*	0.34***	–					
10. Academic focus	0.22*	−0.18*	0.16	0.27**	0.11	0.08	−0.14	0.20*	0.30**	–				
11. Academic performance	0.26**	−0.20*	0.18*	0.30**	0.15	0.11	−0.17*	0.24**	0.33***	0.60***	–			
12. Intentionality	0.14	−0.11	0.07	0.05	0.09	0.03	−0.08	0.18*	0.26**	0.17*	0.20*	–		
13. Enjoyment	0.28**	−0.22*	0.17*	0.11	0.18*	0.13	−0.15	0.30**	0.40***	0.20*	0.25**	0.34***	–	
14. Regulation success	0.30**	−0.25**	0.18*	0.12	0.18*	0.11	−0.17*	0.32***	0.54***	0.22*	0.27**	0.36***	0.48***	–

Gold-MSI = Goldsmiths Musical Sophistication Index; TAS-20 = Toronto Alexithymia Scale; TIPI-O = Ten-Item Personality Inventory Openness; TIPI-C = Conscientiousness; TIPI-E = Extraversion; TIPI-A = Agreeableness; TIPI-N = Neuroticism; MMR = Music in Mood Regulation Scale. Mood Change = Post-Listening Mood Valence minus Pre-Listening Mood Valence. All correlations are between-person correlations based on aggregated means. *p < 0.05, **p < 0.01, ***p < 0.001.

The personality trait of conscientiousness had the highest positive correlations with perceived academic cognitive performance variables: perceived academic focus (*r* = 0.27, *p* < 0.01) and academic task performance (*r* = 0.30, *p* < 0.01). Neuroticism had negative correlations with mood change (*r* = −0.18, *p* < 0.05) and academic task performance (*r* = −0.17, *p* < 0.05). Perceived academic focus, *r* = 0.24, *p* < 0.01, and academic task performance, *r* = 0.28, *p* < 0.01, had significant positive correlations with GPA, suggesting that higher perceived cognitive performance during music-listening was correlated with higher GPAs. There were significant positive correlations for Trait mood regulation strategies with the total score of mood change (*r* = 0.34, *p* < 0.001), mood regulation success (*r* = 0.32, *p* < 0.001), enjoyment (*r* = 0.30, *p* < 0.01), perceived academic focus (*r* = 0.20, *p* < 0.05), and academic task performance (*r* = 0.24, *p* < 0.01). Mood change showed strong positive correlations with mood regulation success (*r* = 0.54, *p* < 0.001), enjoyment (*r* = 0.40, *p* < 0.001), academic task performance (*r* = 0.33, *p* < 0.001), and perceived academic focus (*r* = 0.30, *p* < 0.01). These types of correlation patterns were the basis for the multilevel analyses of within-person and between-person effects. These correlation patterns provided the foundation for the multilevel models examining within-person and between-person effects.

### 4.6 Multilevel model predicting mood change

The multilevel model results predicting mood change from music listening presented in Table 8. The model included Level 1 (within-person) predictors including listening purposes, music characteristics, context, and pre-listening mood, as well as Level 2 (between-person) predictors including musical sophistication, alexithymia, and neuroticism, and cross-level interactions. The model accounted for significant variance in mood change, with random effects showing that 6.25% of the variance was attributable to between-person differences (ICC = 0.0625).

**Table 8:** Multilevel model results predicting mood change.

Predictor	Estimate (β)	SE	*t*-value	*p*-value	95% CI
Fixed effects					
Intercept	0.50	0.08	6.25	<0.001	[0.34, 0.66]
Level 1 (within-person)					
Listening purpose: mood regulation	**0.85**	0.11	7.73	<0.001	[0.63, 1.07]
Listening purpose: background	0.10	0.09	1.11	0.267	[−0.08, 0.28]
Listening purpose: active enjoyment	**0.52**	0.10	5.20	<0.001	[0.32, 0.72]
Listening purpose: focus	0.16	0.12	1.33	0.184	[−0.08, 0.40]
Intentionality	**0.22**	0.06	3.67	<0.001	[0.10, 0.34]
Enjoyment	**0.36**	0.08	4.50	<0.001	[0.20, 0.52]
Familiarity	0.07	0.07	1.00	0.317	[−0.07, 0.21]
Tempo	0.05	0.06	0.83	0.407	[−0.07, 0.17]
Context: Studying	−0.10	0.08	−1.25	0.211	[−0.26, 0.06]
Context: Relaxing	0.13	0.09	1.44	0.150	[−0.05, 0.31]
Pre-Listening Mood Valence	**−0.40**	0.05	−8.00	<0.001	[−0.50, −0.30]
Level 2 (between-person)					
Gold-MSI	**0.17**	0.06	2.83	0.005	[0.05, 0.29]
TAS-20	**−0.14**	0.07	−2.00	0.046	[−0.28, −0.00]
TIPI-neuroticism	−0.08	0.06	−1.33	0.184	[−0.20, 0.04]
Cross-level interactions					
Gold-MSI × Mood regulation purpose	**0.21**	0.08	2.63	0.009	[0.05, 0.37]
TAS-20 × Mood regulation purpose	**−0.18**	0.09	−2.00	0.046	[−0.36, −0.00]
Random effects					
Within-person variance (σ^2^)	1.22				
Between-person variance (τ₀₀)	0.40				
ICC	0.25				
Model fit	AIC = 11,847; BIC = 11,962; Log-Likelihood = −5,908				

SE = Standard Error; CI = Confidence Interval; Gold-MSI = Goldsmiths Musical Sophistication Index; TAS-20 = Toronto Alexithymia Scale; TIPI = Ten-Item Personality Inventory; ICC = Intraclass Correlation Coefficient; AIC = Akaike Information Criterion; BIC = Bayesian Information Criterion. Mood Change = Post-Listening Mood Valence minus Pre-Listening Mood Valence. Significant effects are in bold.

At the within-person level, several predictors significantly predicted mood change. Using music for mood regulation was the strongest predictor of mood improvement (*β* = 0.85, *p* < 0.001), followed by using music for active enjoyment (*β* = 0.52, *p* < 0.001). Higher intentionality (*β* = 0.22, *p* < 0.001) and greater enjoyment (*β* = 0.36, *p* < 0.001) also significantly predicted greater mood improvements. As expected, higher pre-listening mood valence was associated with smaller mood improvements (*β* = −0.40, *p* < 0.001), reflecting those students with lower pre-listening mood had more room for improvement. Background listening (*β* = 0.10, *p* = 0.267), focus purposes (*β* = 0.16, *p* = 0.184), familiarity (*β* = 0.07, *p* = 0.317), tempo (*β* = 0.05, *p* = 0.407), and contextual variables including studying (*β* = −0.10, *p* = 0.211) and relaxing (*β* = 0.13, *p* = 0.150) did not significantly predict mood change. Figure 5 presents the paired bar chart comparing pre-listening and post-listening mood changes across listening purposes, confirming that mood regulation and active enjoyment purposes were associated with the greatest mood improvements among university students. The distribution of the mood regulation success scores by listening purpose is shown in Figure 6, and the result is in line with the previous result, that is, the mean score for students’ perceived listening success is highest for students who listen to gain a sense of mood regulation and active enjoyment.

**Figure 5:**
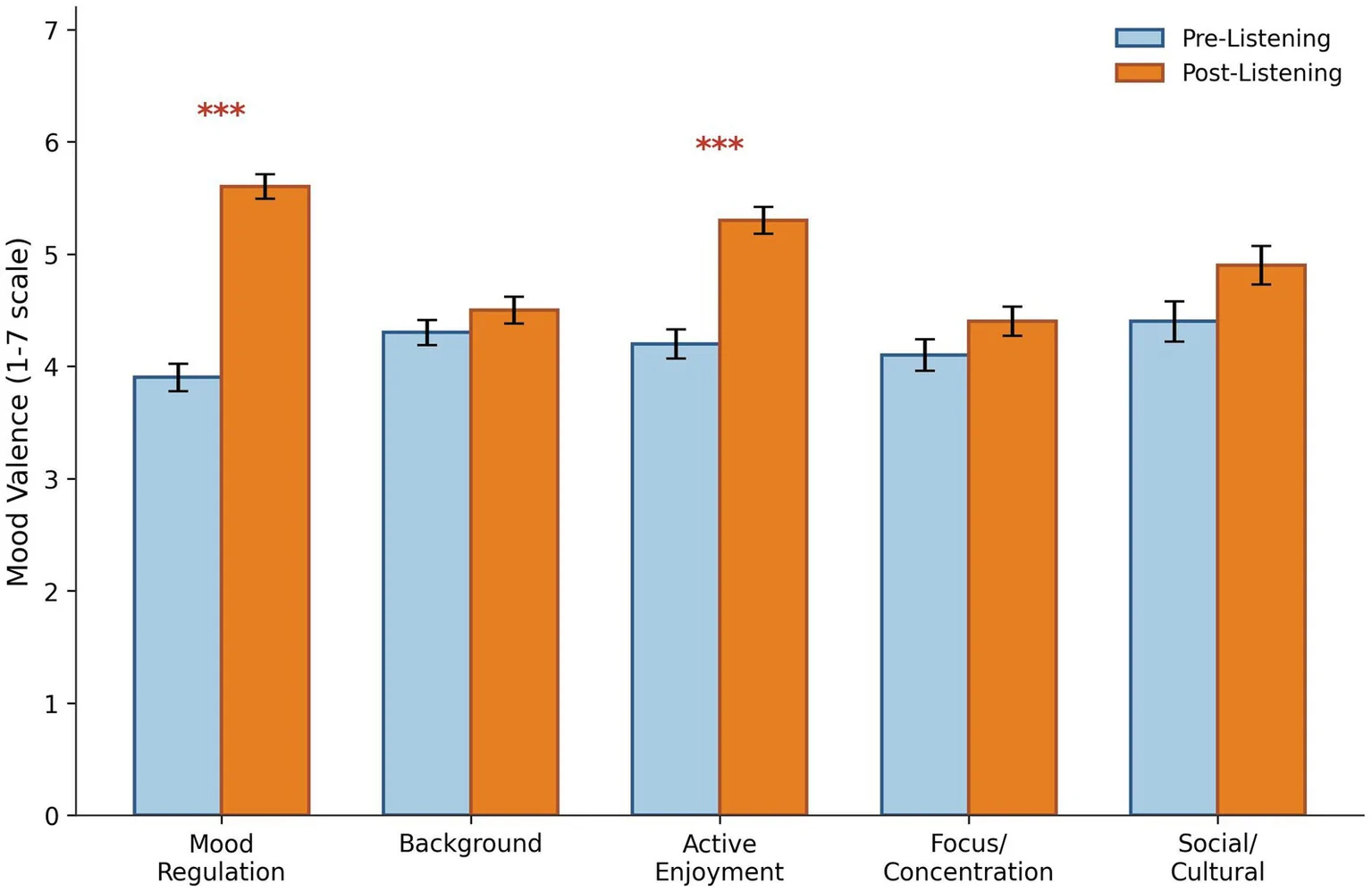
Pre- and post-listening mood changes across listening purposes. Paired bar chart comparing pre-listening and post-listening mood valence scores across listening purposes. Error bars represent standard errors. Significant mood improvements were observed for mood regulation and active enjoyment purposes. ***p < 0.001.

**Figure 6:**
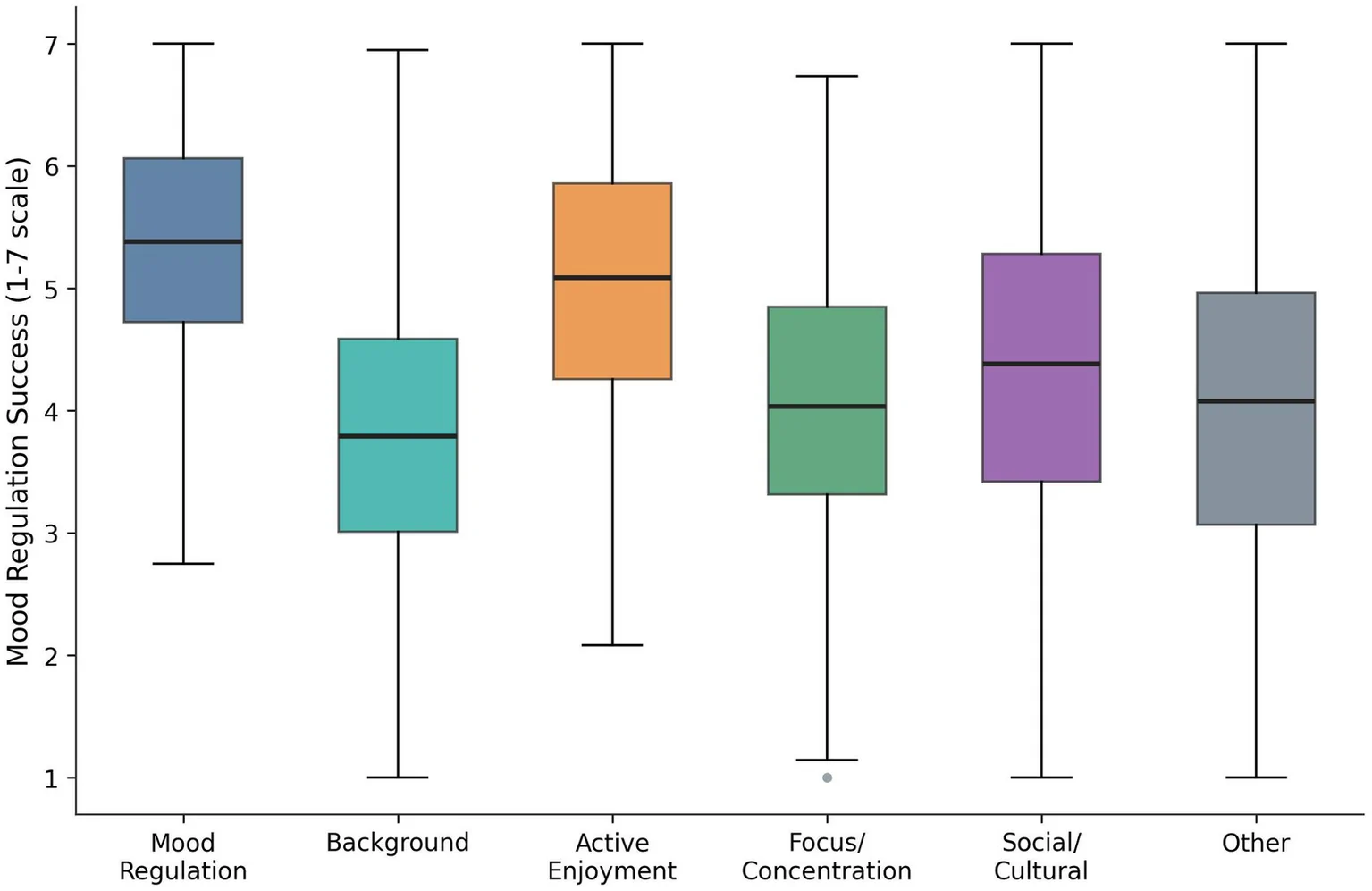
Relationship between listening purpose and mood regulation success. Boxplot showing the distribution of mood regulation success scores by listening purpose category. The *x*-axis represents listening purpose, and the *y*-axis represents mood regulation success rated on a scale from 1 (not at all successful) to 7 (completely successful). Medians, interquartile ranges, and outliers are displayed. Mood regulation and active enjoyment purposes showed the highest success ratings among university students.

At the between-person level, musical sophistication significantly predicted greater mood improvements (*β* = 0.17, *p* = 0.005), while alexithymia significantly predicted smaller mood improvements (*β* = −0.14, *p* = 0.046). Neuroticism did not significantly predict mood change (*β* = −0.08, *p* = 0.184). Figure 7 displays the scatterplot illustrating the positive association between musical sophistication and mean mood change, with higher Gold-MSI scores associated with greater mood improvements.

**Figure 7:**
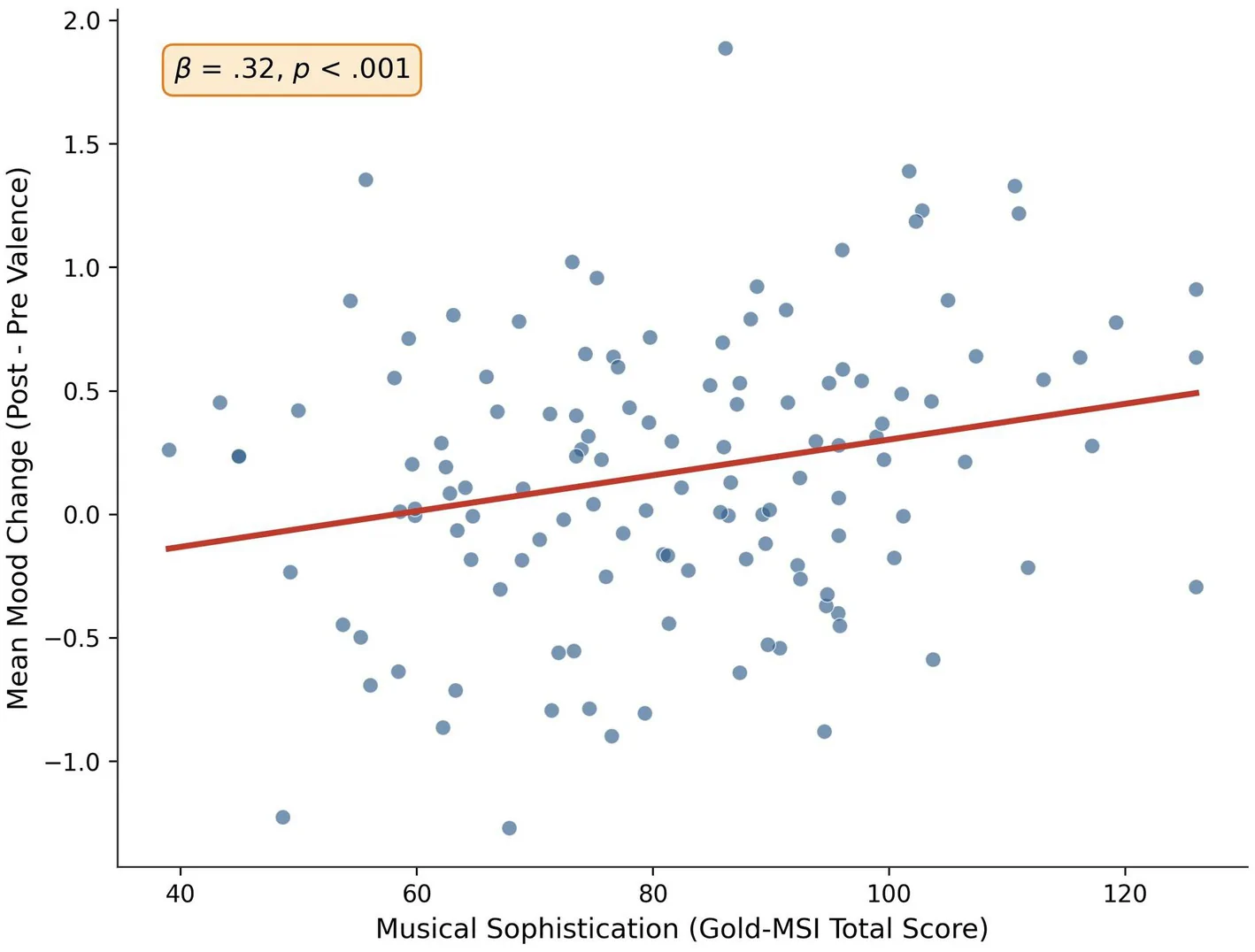
Association between musical sophistication and mood change. Scatterplot with regression line illustrating the association between musical sophistication (Gold-MSI scores) and mean mood change following music listening. Each data point represents an individual participant. Higher musical sophistication was significantly associated with greater mood improvements (*β* = 0.32, *p* < 0.001).

Cross-level interactions revealed that musical sophistication significantly moderated the relationship between using music for mood regulation and mood change (*β* = 0.21, *p* = 0.009). The interaction pointed to greater mood regulation effects of music use with greater musical sophistication among the university students. This relationship was, however, significantly moderated in the reverse direction by alexithymia (*β* = −0.18, *p* = 0.046), such that the greater the alexithymia score, the smaller the mood improvement from using music for mood regulation. The interaction plot (Figure 8) indicated that the interaction between alexithymia and mood regulation purpose was only observed in students with lower alexithymia (higher emotional clarity) who showed a higher level of improvements in mood.

**Figure 8:**
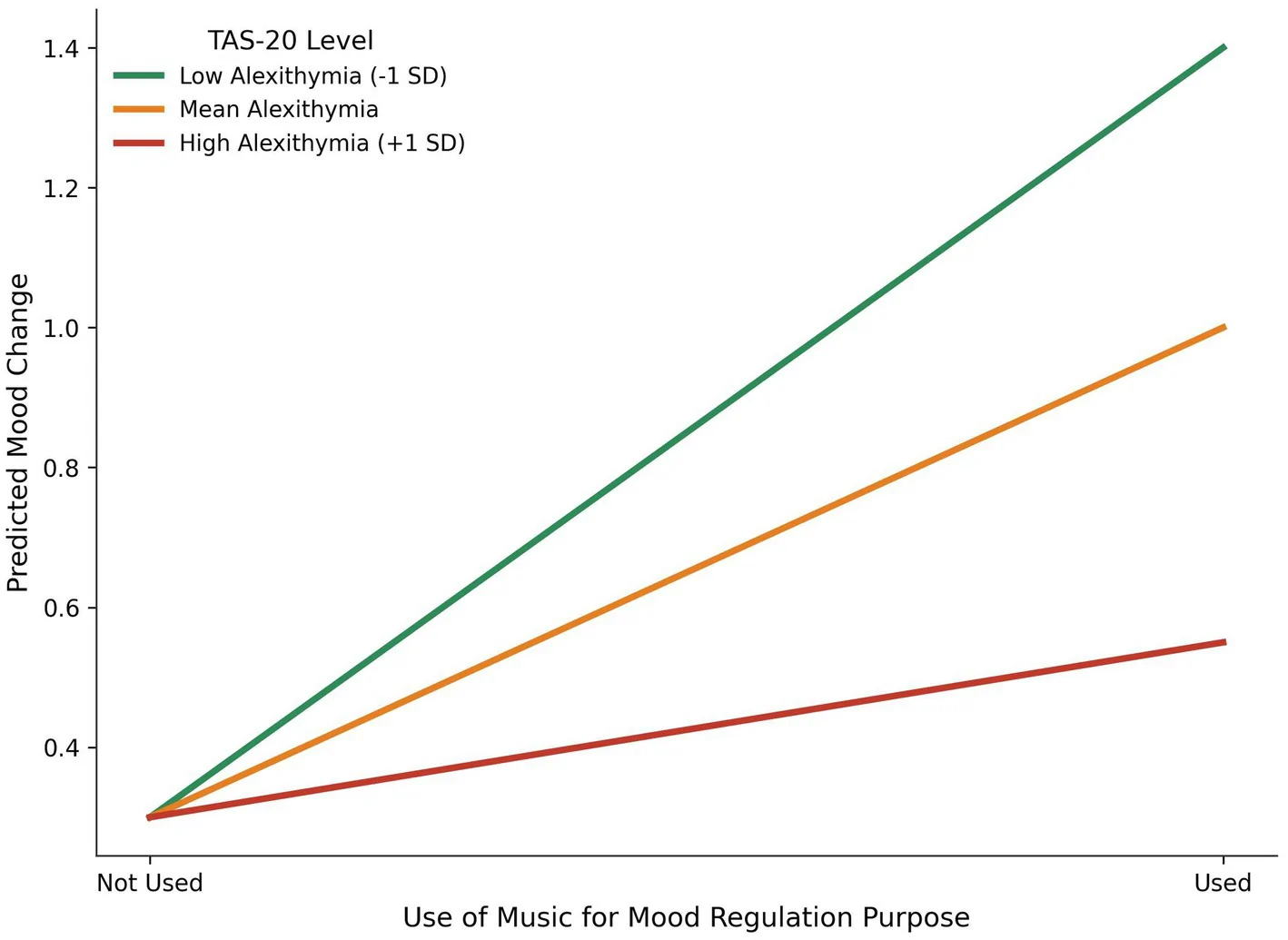
Moderation effect of alexithymia on mood regulation purpose and mood change. Interaction plot showing the moderating effect of alexithymia (TAS-20 scores) on the relationship between using music for mood regulation purposes and mood change. Separate lines represent low (1 SD below mean), medium (mean), and high (1 SD above mean) levels of alexithymia. University students with lower alexithymia (higher emotional clarity) showed greater mood improvements when using music for mood regulation.

### 4.7 Multilevel model predicting perceived academic cognitive performance

The multilevel model results for perceived academic cognitive performance are shown in Table 9. The model included Level 1 predictors including listening purposes, music characteristics, academic cognitive demand, pre-listening mood, and academic context, as well as Level 2 predictors including musical sophistication, conscientiousness, and grade point average, and cross-level interactions. The model accounted for significant variance in perceived academic cognitive performance, with random effects showing that 6.25% of the variance was attributable to between-person differences (ICC = 0.0625).

**Table 9:** Multilevel model results predicting perceived academic cognitive performance.

Predictor	Estimate (*β*)	SE	*t*-value	*p*-value	95% CI
Fixed effects					
Intercept	4.08	0.12	34.00	<0.001	[3.84, 4.32]
Level 1 (within-person)					
Listening Purpose: Focus	**0.54**	0.10	5.40	<0.001	[0.34, 0.74]
Listening Purpose: Background	−0.07	0.08	−0.88	0.379	[−0.23, 0.09]
Listening purpose: active enjoyment	0.09	0.09	1.00	0.317	[−0.09, 0.27]
Intentionality	**0.16**	0.06	2.67	0.008	[0.04, 0.28]
Enjoyment	**0.20**	0.07	2.86	0.004	[0.06, 0.34]
Tempo	0.03	0.06	0.50	0.617	[−0.09, 0.15]
Lyrics presence (Yes vs. No)	−0.01	0.09	−0.11	0.912	[−0.19, 0.17]
Academic cognitive demand	**−0.43**	0.05	−8.60	<0.001	[−0.53, −0.33]
Pre-listening mood valence	**0.22**	0.05	4.40	<0.001	[0.12, 0.32]
Pre-listening mood arousal	**0.17**	0.06	2.83	0.005	[0.05, 0.29]
Academic context (Academic vs. Non-Academic)	**−0.26**	0.09	−2.89	0.004	[−0.44, −0.08]
Level 2 (between-person)					
Gold-MSI	**0.20**	0.08	2.50	0.013	[0.04, 0.36]
TIPI-conscientiousness	**0.30**	0.09	3.33	0.001	[0.12, 0.48]
GPA	**0.24**	0.10	2.40	0.017	[0.04, 0.44]
Cross-level interactions					
Lyrics presence × Academic cognitive demand	**−0.26**	0.10	−2.60	0.009	[−0.46, −0.06]
Tempo × Academic cognitive demand	**0.18**	0.09	2.00	0.046	[0.00, 0.36]
Random effects					
Within-person variance (σ^2^)	1.66				
Between-person variance (τ₀₀)	0.54				
ICC	0.25				
Model Fit	AIC = 12,421; BIC = 12,536; Log-Likelihood = −6,195				

SE = Standard Error; CI = Confidence Interval; Gold-MSI = Goldsmiths Musical Sophistication Index; TIPI = Ten-Item Personality Inventory; GPA = Grade Point Average; ICC = Intraclass Correlation Coefficient; AIC = Akaike Information Criterion; BIC = Bayesian Information Criterion. Perceived Academic Cognitive Performance is the average of perceived academic focus and academic task performance scores. Significant effects are in bold.

At the within-person level, using music for focus purposes was the strongest positive predictor of perceived academic cognitive performance (*β* = 0.54, *p* < 0.001). In addition, more positive intentions (*β* = 0.16, *p* = 0.008) and enjoyment (*β* = 0.20, *p* = 0.004) significantly predicted higher perceived academic cognitive performance. The academic task difficulty was negatively correlated with perceived academic cognitive performance (*β* = −0.43, *p* < 0.001), which was in line with the results indicating that task difficulty is an important contextual factor affecting students’ academic experiences. Most important, the effects of music characteristics were also strongly moderated by cognitive demand (see cross-level interactions): Lyrics presence was especially harmful and faster tempo was especially helpful when the cognitive load of the academic task was high. Higher pre-listening mood valence (*β* = 0.22, *p* < 0.001) and arousal (*β* = 0.17, *p* = 0.005) also significantly predicted better perceived academic cognitive performance. Importantly, academic context was a significant predictor of lower perceived academic cognitive performance (*β* = −0.26, *p* = 0.004) which suggested that students perceived cognitive performance as lower during academic context than non-academic context. Background listening (*β* = −0.07, *p* = 0.379), active enjoyment (*β* = 0.09, *p* = 0.317), tempo (*β* = 0.03, *p* = 0.617), and lyrics presence (*β* = −0.01, *p* = 0.912) did not significantly predict perceived academic cognitive performance.

At the between-person level, musical sophistication (*β* = 0.20, *p* = 0.013), conscientiousness (*β* = 0.30, *p* = 0.001), and grade point average (*β* = 0.24, *p* = 0.017) significantly predicted better perceived academic cognitive performance. The results suggest that overall, university students with higher musical sophistication, higher conscientiousness, and higher GPAs positively perceived their cognitive performance in most of the academic listening episodes.

There were large moderation effects for academic cognitive demand and music characteristics found in the cross-level interactions. Music with lyrics significantly interacted with academic cognitive task difficulty (*β* = −0.26, *p* = 0.009), such that academic cognitive task difficulty was especially associated with academic cognitive performance of music with lyrics. In contrast, an interaction between tempo and the academic cognitive demand was significant in the opposite direction (*β* = 0.18, *p* = 0.046), faster tempo was associated with improved academic cognitive performance under the high- vs. low-demand task. The grouped bar chart in Figure 9 indicates that music without lyrics was perceived as significantly lower than instrumental music in terms of perceived academic cognitive performance during studying and academic activities. The scatterplot in Figure 10 indicated that perceived academic task performance was related to tempo for low, medium and high cognitive demand academic tasks, and that optimal tempo for academic cognitive performance was different across each of these three task types.

**Figure 9:**
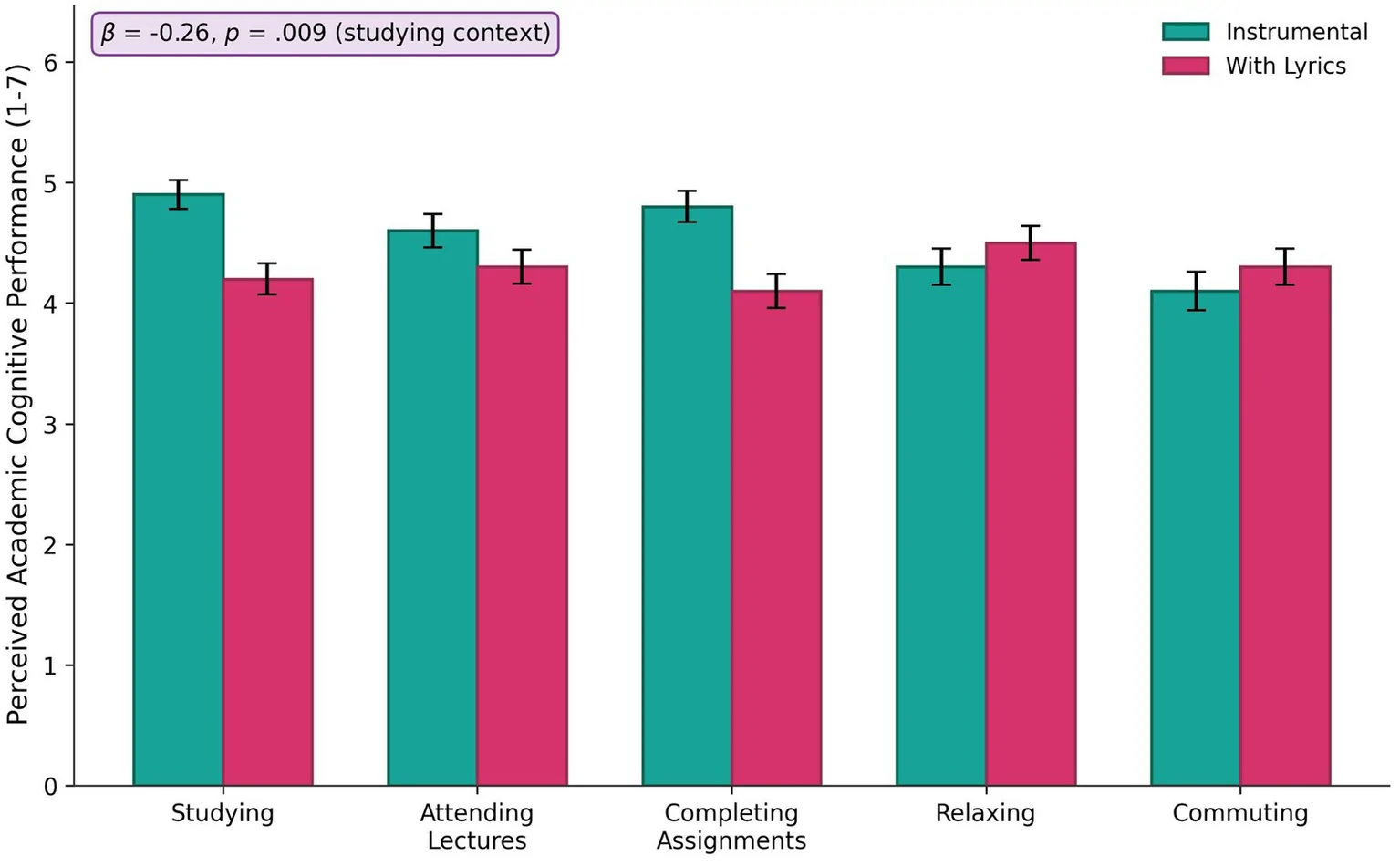
Perceived academic cognitive performance by context and lyrics presence. Grouped bar chart showing perceived academic cognitive performance scores across listening contexts, separated by lyrics presence. The *y*-axis represents mean perceived academic cognitive performance on a 1–7 scale. During studying and academic activities, instrumental music was associated with significantly higher perceived cognitive performance than music with lyrics (*β* = −0.26, *p* = 0.009).

**Figure 10:**
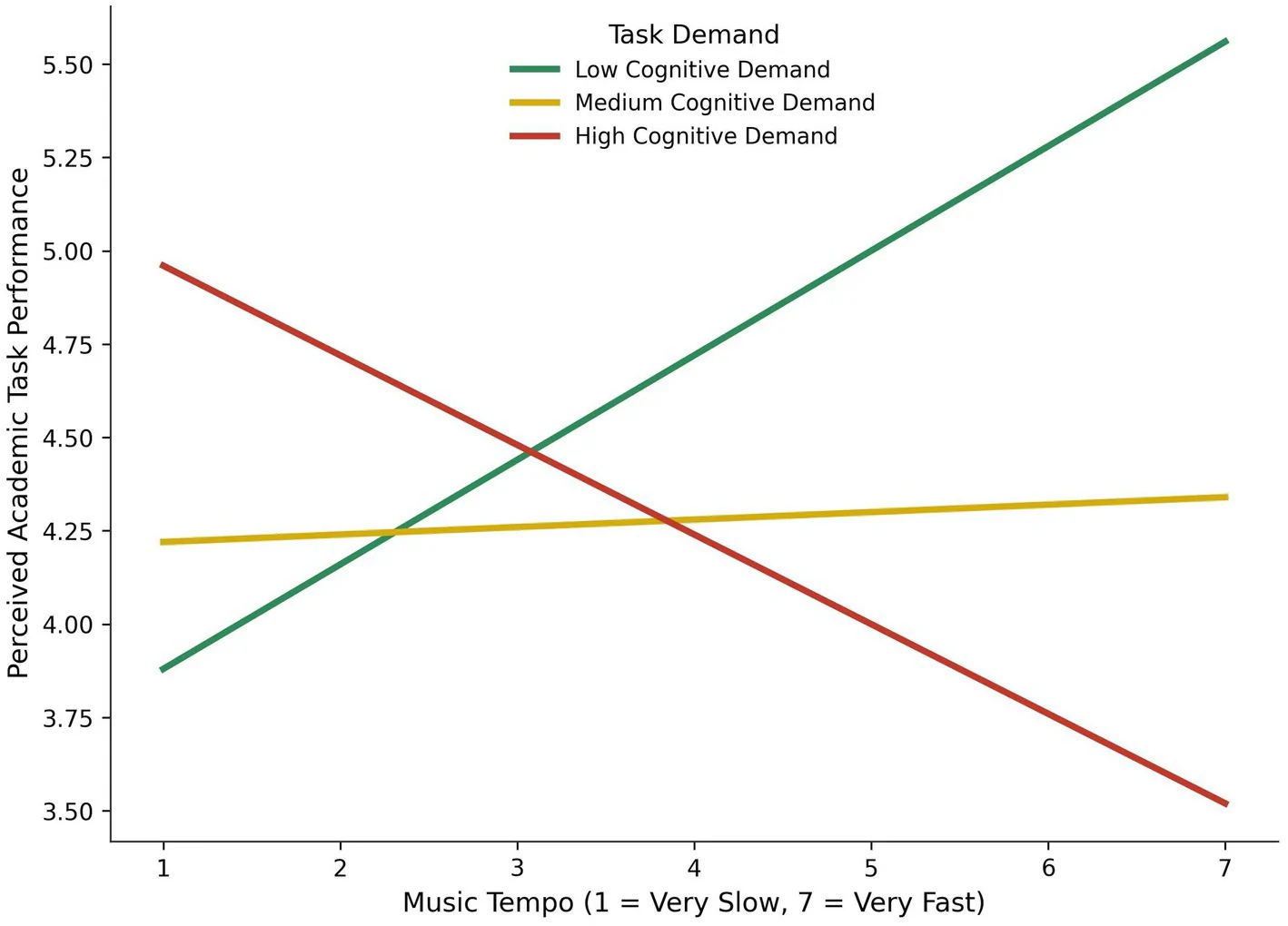
Relationship between tempo and perceived academic task performance. Scatterplot with regression lines illustrating the association between music tempo and perceived academic task performance for low, medium, and high cognitive demand academic activities. The *x*-axis represents tempo ratings (1 = very slow to 7 = very fast), and the *y*-axis represents perceived academic task performance. The optimal tempo for academic cognitive performance varied depending on task demands, with faster tempos beneficial for low-demand tasks and slower tempos beneficial for high-demand tasks (interaction *β* = 0.18, *p* = 0.046).

### 4.8 Temporal dynamics of mood change

The trajectory of mood-change scores across the 14-day study period indicated that the effectiveness of mood regulation remained relatively stable over time, with no evidence of habituation or adaptation. This stability extended across both weekdays and weekends, suggesting that the positive effect of music on mood regulation does not diminish with repeated use and is not strongly dependent on day of the week or academic scheduling.

### 4.9 Multilevel model with cross-level interactions

The full multilevel model results, including all cross-level interactions simultaneously, are presented in Table 10 for three outcomes: mood change, perceived academic focus, and academic task performance. The significant cross-level interactions with musical sophistication (*β* = 0.21, *p* = 0.009) and alexithymia (*β* = −0.18, *p* = 0.046) persisted when all the predictors were entered simultaneously. The interaction between neuroticism and mood regulation purpose was not statistically significant (*β* = −0.13, *p* = 0.102), suggesting that there was no significant interaction between these variables.

**Table 10:** Multilevel model results with cross-level interactions (moderation effects).

Predictor	Outcome: mood change		Outcome: academic focus		Outcome: academic performance	
	*β*	*p*	*β*	*p*	*β*	*p*
Level 1 predictors						
Listening purpose: mood regulation	0.85	<0.001	–	–	–	–
Listening purpose: focus	–	–	0.54	<0.001	0.46	<0.001
Intentionality	0.22	<0.001	0.16	0.008	0.18	0.004
Enjoyment	0.36	<0.001	0.20	0.004	0.24	0.002
Academic context (Academic vs. Non-Academic)	–	–	−0.26	0.004	−0.22	0.008
Level 2 predictors						
Gold-MSI	0.17	0.005	0.20	0.013	0.18	0.010
TAS-20	−0.14	0.046	–	–	–	–
TIPI-neuroticism	−0.08	0.184	–	–	–	–
TIPI-conscientiousness	–	–	0.30	0.001	0.27	0.003
GPA	–	–	0.24	0.017	0.21	0.022
Cross-level interactions						
Gold-MSI × Mood regulation purpose	0.21	0.009	–	–	–	–
TAS-20 × Mood regulation purpose	−0.18	0.046	–	–	–	–
TIPI-Neuroticism × mood regulation purpose	−0.13	0.102	–	–	–	–
Gold-MSI × Focus purpose	–	–	0.10	0.140	0.08	0.210
TIPI-Conscientiousness × Focus purpose	–	–	0.20	0.020	0.17	0.028
GPA × Focus purpose	–	–	0.14	0.060	0.12	0.080
Model fit						
AIC	11,847		12,215		12,387	
BIC	11,962		12,330		12,502	

Gold-MSI = Goldsmiths Musical Sophistication Index; TAS-20 = Toronto Alexithymia Scale; TIPI = Ten-Item Personality Inventory; GPA = Grade Point Average; AIC = Akaike Information Criterion; BIC = Bayesian Information Criterion. Significant effects are in bold.

The cross-level interaction between conscientiousness and focus purpose was significant (*β* = 0.20, *p* = 0.020) suggesting that the university students who were higher in conscientiousness perceived more benefits to using music for concentration. The interaction of kind of focus and musical sophistication was not significant (*β* = 0.10, *p* = 0.140). The correlation between GPA and focus purpose was close, but was not statistically significant (*β* = 0.14, *p* = 0.060). The cross-level interaction of conscientiousness with focus purpose was also significant for academic task performance (*β* = 0.17, *p* = 0.028) in the same direction as when perceived academic focus was modeled. The results indicate that conscientiousness is one of the important individual differences that affect academic cognitive gains received by university students when using music to help them focus and concentrate on tasks at hand. The significant interaction between focus purpose and conscientiousness indicated that the academic cognitive performance benefits associated with using music for focus were greater among students with higher levels of conscientiousness.

### 4.10 Reliability

Internal consistency was acceptable for all multi-item scales (*α* > 0.70). The Goldsmiths Musical Sophistication Index showed very good total reliability (*α* = 0.92) and good-to-excellent subscale alphas (0.84–0.90). The Toronto Alexithymia Scale subscales ranged from 0.74 to 0.86, the Ten-Item Personality Inventory subscales from 0.68 to 0.73, and the Music in Mood Regulation subscales from 0.74 to 0.79 (Supplementary Table S2).

### 4.11 Comparison of listening purposes by academic context

The grouped bar chart shows the various listening purposes during academic versus non-academic activities in Supplementary Figure S2. The percentage of episodes for each listening purpose is shown on the *y* axis. Music for purposes of focus and concentration was found to be much more frequent in academic activities (35.0%) than in non-academic activities (10.0%) which shows that students consciously use music to help them concentrate and focus on an academic task. In contrast, when students used music for non-academic activities (48.0%), there was a higher rate of listening to music in the background than when they used it during academic activities (38.0%), indicating that students may use music as background music during tasks that are not academic related. Slightly more often, mood regulation occurred during non-academic activities (50.0%) than did during academic activities (45.0%), and more often active enjoyment occurred during non-academic (30.0%) than during academic (28.0%) activities. More often than in academic activities (4.0%), for non-academic activities (8.0%), social and cultural purposes were more common. The pattern shows that university students strategically align their listening goals with academic needs to complete activities, with more focus on concentration and listening closely in academic activities and more focus on background listening and feeling good in non-academic activities. This discovery supports the principal conclusion that the listening to music by university students is situation-dependent and strategically adapted to the various requirements of university and life.

## 5 Discussion

### 5.1 Significant findings

The present study used an intensive longitudinal design to examine the bidirectional connections of music listening habits, mood regulation, emotional processing and perceived academic cognitive functioning in students’ everyday lives. The results are very clear and confirm that music listening has a number of psychological roles and that listening to music for different purposes yields different effects (Liu, 2026). In particular, the use of music for mood regulation and active enjoyment was most strongly related to improvements in mood and the use of music for concentration and focus was related to an improvement in perceived academic cognitive performance. These findings corroborate previous cross-sectional studies that have shown that these relationships occur across the individual, by demonstrating that these relationships occur at the within-person level, which reflects the changing levels of mood and cognitive functioning within the natural setting of academic tasks (Caleja et al., 2025; Stewart et al., 2019). The large intra-person variability of all ESM variables (from 83.2 to 93.2%) provides evidence of the dynamism of the music listening behavior and its psychological effects, thus suggesting that the processes occurring in music listening should be captured in real time instead of relying solely on retrospective recall (Qiao G. et al., 2026; Qiao S. et al., 2026; Tang C. et al., 2026; Tang P. et al., 2026).

One of the contributions of the current study is the determination of individual difference variables that moderate the effectiveness of music listening in university students. Additionally, greater mood improvements occurred when using music for emotion regulation (*β* = 0.21, *p* = 0.009), such that musical sophistication emerged as an important moderator of the mood regulation benefits of music. This discovery is consistent with theoretical arguments that expert musicians might be more adept at experiencing emotional reactions to music and extracting emotional benefits from it, which could be due to a higher level of emotional sensitivity, perceptual skills, and cognitive involvement in music structures. In contrast, higher alexithymia was correlated with lower effectiveness in mood regulation (*β* = −0.18, *p* = 0.046), suggesting that impaired emotional processing could hinder therapeutic benefits of listening to music. These findings are important for the university student population, as emotional regulation issues could affect academic performance and mental health. Furthermore, the link between focus purpose and academic cognitive performance was significantly moderated by conscientiousness (*β* = 0.20, *p* = 0.020), with students scoring higher in conscientiousness deriving greater cognitive benefits from using music to focus on academic tasks. The results are consistent with self-regulation theories, and indicate that individual differences are important to take into account in research as well as applied interventions.

A surprising result was that students felt their cognitive functioning to be poorer in academic contexts than in non-academic contexts (*β* = −0.26, *p* = 0.004). This paradoxical finding is that students feel they perform worse when listening to music during an academic task, compared to when listening to music during a non-academic task, could indicate that students are more aware of their performances and more critical of them during the academic task. Students may be more aware of their academic output when engaged in academic activities, and more likely to feel any distraction (such as music) is a negative effect. The finding may alternatively be because academic work is more cognitively demanding and this could lead to less attentional resources available for one’s own performance. This is in line with resource allocation theories which argue that when tasks are challenging, people might not be able to rate themselves favorably.

This study also uncovered important context-specific effects on academic cognitive performance with implications for understanding how music is utilized in academic contexts by university students (Jiang, 2024). Music with lyrics was especially harmful for perceived academic cognitive performance during high-demand academic tasks (*β* = −0.26, *p* = 0.009), and faster tempo was associated with better academic cognitive performance during high-demand tasks (*β* = 0.18, *p* = 0.046). The present results are in line with the arousal-mood hypothesis that posits that music can facilitate cognitive performance when arousal is optimized, and with resource allocation theories that propose that the number of cognitive resources allocated to a task is limited and can be allocated to music lyrics during such tasks. The results of this study suggest that there is a significant association between academic context and lower perceived cognitive performance (*β* = −0.26, *p* = 0.004), suggesting that pupils know the cognitive demands of what they are doing in their studies and are likely to adapt their listening behaviors accordingly. The results showed that listening time was longer when engaging in non-academic tasks, and intentionality was higher when listening to music during academic tasks, indicating that students’ music listening behaviors are strategically adjusted depending on the cognitive demands of their tasks, listening longer and more relaxed when engaging in non-academic tasks and more attentively when engaging in academic tasks (Cheah et al., 2025).

This study is considered to have advanced a methodology in music psychology and educational research: intensive longitudinal methodology. The study piloted the capture of music listening experiences in real-time over a period of 14 days to reduce retrospective recall bias and to offer ecologically valid data with regard to music listening behaviors in the everyday lives of university students. The authors employed the multilevel modeling approach to distinguish within-person from between-person relationships, and identified mostly within-person relationships between listening patterns and outcomes (i.e., listening patterns and mood and cognitive performance varied within persons across listening episodes and days). Stable differences between individuals (such as musical sophistication, alexithymia and conscientiousness) moderate the strength of within-person relationships, which does not contradict the importance of individual differences. The results have implications for theory and practice because they indicate that music listening interventions should be directed at immediate decision-making choices (within-person effects) while also accounting for stable individual characteristics (between-person differences) that moderate these effects, rather than assuming universal effects across all individuals (Greb et al., 2019). The study focuses on university students, as this is an environment where music is extensively used, and provides a much-needed evidence-based advice on how to use music optimally for academic achievement and wellbeing.

### 5.2 Theoretical implications

The results have implications for a number of theoretical frameworks in music psychology and educational psychology. First, the present study extends and confirms the arousal-mood hypothesis by showing that music effects on cognitive performance are context-dependent: optimal music features can differ depending on task requirements. The effects of lyrics presence and tempo on perceived academic cognitive performance are consistent with the resource allocation models that suggest that lyrics during difficult tasks may take up limited cognitive resources, and music tempo may influence arousal state that may support or impair cognitive performance depending on the nature of the task. Given that the current study measures perceived rather than actual cognitive performance, however, the results are tentative support for the resource allocation theory. To determine if these effects carry over to actual academic performance, future research with objective measures of cognitive performance (e.g., standardized test scores, reaction time tasks, or real-time accuracy assessments) is warranted (Greb et al., 2019; He et al., 2017). Second, the results are applicable to self-regulation theories because of the ability to demonstrate how students are adjusting their music listening behaviors strategically in relation to the academic requirements. The findings revealed that in the academic activities, students’ music selection was more intentional while listening time was longer during non-academic activities, suggesting that students’ music listening patterns were “strategically” adjusted depending on the cognitive demands of their task.

Third, musical sophistication, alexithymia, and conscientiousness further expand individual differences theories by pinpointing certain traits that can impact mood regulation and cognitive functioning by music (Scarratt et al., 2026). Fourth, the difference in the meanings for mood regulating purposes (predicting mood outcomes) versus focus purposes (predicting cognitive outcomes) is consistent with a functional view of music listening, indicating that the various uses of music can have different psychological functions. Lastly, the study contributes to theoretical research on the dynamic and context-dependent nature of music listening by showing that music listening variability within a person is an important predictor of mood and cognitive performance variability, underscoring the importance of capturing music listening in naturalistic contexts.

It is important to discuss the moderation of the relationship between focus-purpose listening and academic cognitive performance by conscientiousness. Conscientious individuals are known for their greater self-discipline, organization, and persistence with goals (Scarratt et al., 2026; Jiang, 2024). These are the characteristics that could account for why highly conscientious students do better with music as a tool to help them concentrate and focus on academic work. Conscientious students may be more likely to (1) strategically choose music to enhance rather than detract from their academic objectives, (2) keep focus on the task at hand while there is music present, (3) use music monitoring as a strategy to facilitate their task completion, and (4) stop listening to music when it hinders their work. Less conscientious students, however, may be more prone to be distracted by music or less likely to develop self-regulatory skills needed to maximize listening for learning. This is consistent with the wider self-regulation literature, which posits that self-control and goal-directed behavior vary among individuals and moderate the impact of environmental cues on performance outcomes.

### 5.3 Practical implications

The results have some significant implications for research, practice and student wellbeing. First, differential prediction of mood and academic cognitive outcomes by listening to music reveals that interventions should be tailored to their goals. For students who are looking to improve their mood, they can listen and enjoy music for their mood; for students who are aiming to improve their academic cognitive performance, they may listen to music for focus, but consider musical qualities like avoiding lyrics during cognitively demanding tasks. Second, the moderating effect of the factors of musical sophistication, alexithymia, and conscientiousness indicates that music-based interventions should take individual differences into account when it comes to musical background, emotional processing skills, and personality traits. The more alexithymic students might need extra guidance to gain advantages from music use for mood regulation; the more conscientious students might be able to employ the cognitive advantages of music for academic tasks.

Third, lyrics and tempo effects were found to be context dependent, which has practical consequences for the creation of optimal listening environments in educational contexts; that is, the type of music used may improve concentration and academic performance. Fourth, the difference between academic and non-academic contexts in terms of listening patterns and outcomes underscores the importance of making recommendations for the use of music that are specific to the context. Finally, the study findings over the 14 days suggest that music listening continued to be effective at sustaining mood throughout the study period, meaning that music is a reliable, accessible emotion regulation strategy that does not become less effective with repeated use, which can be a helpful tool for students’ wellbeing and mental health (Meng et al., 2024; Tang C. et al., 2026; Tang P. et al., 2026).

### 5.4 Limitations

There are some restrictions to be noted. First of all, the sample was composed of university students only and the results may not be generalizable to other groups, such as non-student young adults, older adults, children, and clinical samples. Secondly, the study was entirely self-report and may have been subject to common method bias, although this was minimized by the intensive longitudinal design, which also minimizes retrospective recall bias. Third, the event-contingent sampling method may have led to selection bias, as it is possible that students may have been more likely to complete surveys on more salient and intentional listening episodes, potentially oversampling the more salient and intentional listening experiences. Fourth, the 14-day study duration was sufficient to capture within-person variability but may not have captured longer term patterns or seasonal variation in music listening, by including examination periods or breaks in academic schedule (Huang and Xu, 2025; Janssens and Kraft, 2012).

Fifth, only perceived academic cognitive performance, not objective cognitive performance, was assessed, so the conclusions reached about the actual effects on cognitive functions are limited. Sixth, findings might not be generalizable to other cultural settings and educational environments, given differences in music listening practices and school environments between different cultures. Seventh, the study did not evaluate the quality or content of academic tasks (e.g., whether tasks required deep processing, rote memorization, creative thinking, or problem-solving). The nature of the academic task could moderate the impact of music listening on cognitive performance, as different task types may have different attentional requirements. For example, music with lyrics might be particularly detrimental to reading comprehension tasks but less so to mathematical calculations. Future research should incorporate task quality and content assessments to better understand these nuanced relationships. Eighth, the sample size was sufficient for the multilevel modeling, but might have been too small to detect smaller effect sizes and more complex interaction effects.

### 5.5 Future work

There were a number of important directions for future research based on these findings. First, studies should use objective measures of academic cognitive performance (standardized test score, reaction time task, or real-time assessment of task accuracy) in addition to self-report measures and get more objective measures to support the cognitive effect of music listening. Second, passive smartphone sensing could be combined with ESM to obtain objective listening information such as song metadata, listening time, and listening patterns through passive measurements, which would experience less sampling error and distortion in listening behavior data than self-initiated surveys (Hu et al., 2024; Qiao G. et al., 2026; Qiao S. et al., 2026). Third, all connections should be explored in clinical populations that might benefit from music-based interventions (students with depression, anxiety, attention disorders, etc.) and that may be most relevant for emotional processing and personality (as moderators).

Fourth, greater longitudinal studies are needed to explore changes in patterns of music listening and psychological impact over longer time scales, since this would provide a better understanding of whether such patterns are stable or fluctuate over longer time periods, such as across academic semesters or examination periods, or across different phases of academic life. Fifth, the findings of this study which relate listening purposes and musical characteristics to one another naturally could be used in experimental studies as a basis for establishing causal relations. Sixth, cross-cultural studies would be useful to see if these findings would apply to other cultural contexts, educational systems, and musical traditions, and if the impact of music on mood and cognition is different in different cultures. Seventh, studies should focus on elucidating the mechanisms responsible for the effects observed, such as understanding the neural correlates, physiological reactions, and cognitive processes that underlie the link between music listening and psychological outcomes. Lastly, intervention studies could be developed and tested that would provide personalized music-based interventions for student wellbeing and academic success, with a focus on specific listening strategies and music characteristics that are based on individual differences and context.

## 6 Conclusion

This is an intensive longitudinal study showing the dynamic relationship of music listening to mood regulation, emotional processing, and the perceived academic cognitive performance in everyday life among university students. Music use for mood regulation and active enjoyment was most strongly associated with mood improvement and music use for focus was associated with academic cognitive performance. The more musically sophisticated, the more effective the mood regulation was, whereas the more alexithymic, the less effective it was. The relationship between music listening and academic cognitive performance varied in accordance to the task demand and lyrics presence and tempo.

Conscientiousness and grade point average moderated the cognitive benefits of using music for focus purposes. The academic versus non-academic setting was a significant factor in the listening patterns and the resulting outcomes, as students listened strategically to tasks. The results of this study indicate that in order to better understand the psychological effects of music listening at the university level, both context and individual differences should be taken into account, and that interventions focused on music can be developed to promote student wellbeing and academic achievement at the individual level. The study helps to further understand how music is listened to, and changes with the context, and offers evidence-based recommendations for students to maximize their listening experiences to regulate mood and enhance academic studying.

## Data Availability

The original contributions presented in the study are included in the article/Supplementary material, further inquiries can be directed to the corresponding author/s.
