# Supplementary material for: Music listening patterns and their associations with mood regulation, emotional processing, and cognitive performance: an intensive longitudinal questionnaire study
Source: Front Psychol. 2026 Sep 24;17:1950103. doi: 10.3389/fpsyg.2026.1950103 (PMC13645529; doi:10.3389/fpsyg.2026.1950103)
Supplement: Supplementary file 1 [file Supplementary_File_1.DOCX]

**SUPPLEMENTARY**

**Music Listening Patterns and Their Associations with Mood Regulation, Emotional Processing, and Cognitive Performance: An Intensive Longitudinal Questionnaire Study**

**Table S1: Complete List of ESM Survey Items and Response Options**

| **Section** | **Item** | **Response Options** |
| --- | --- | --- |
| **Contextual Information** | What time did the listening begin? | Timestamp captured automatically |
|  | Where are you currently? | Home, Campus/Library, Commuting, Exercise, Social Setting, Other |
|  | What is your primary activity? | Studying, Attending Lectures, Completing Assignments, Commuting, Exercising, Relaxing, Socializing, Other |
|  | Is this an academic activity? | Yes, No |
|  | Who are you with? | Alone, With Friends, With Classmates, With Family, With Partner, In Public, Other |
| **Music Characteristics** | What genre(s) best describe the music? | Pop, Rock, Classical, Electronic/Dance, Hip-Hop/Rap, Jazz, Folk/Acoustic, Metal, R&B/Soul, Other |
|  | How familiar is this music to you? | 1 (Not at all familiar) to 7 (Very familiar) |
|  | How much do you enjoy this music? | 1 (Not at all) to 7 (Very much) |
|  | Does the music contain lyrics? | Yes, No (Instrumental), Mixed |
|  | How would you describe the tempo? | 1 (Very slow) to 7 (Very fast) |
|  | How did you select this music? | My Playlist, Algorithm Recommendation, Friend/Other Person, Radio, Other |
| **Listening Purpose** | Why did you choose to listen to music? | Mood Regulation, Background, Active Enjoyment, Focus/Concentration, Social/Cultural, Other (select all that apply) |
| **Intentionality** | How deliberate was your choice of music? | 1 (Not at all deliberate) to 7 (Very deliberate) |
| **Pre-Listening Mood** | Before listening, how did you feel? (Positive) | 1 (Not at all positive) to 7 (Very positive) |
|  | Before listening, how did you feel? (Energetic) | 1 (Not at all energetic) to 7 (Very energetic) |
| **Post-Listening Mood** | Right now, how do you feel? (Positive) | 1 (Not at all positive) to 7 (Very positive) |
|  | Right now, how do you feel? (Energetic) | 1 (Not at all energetic) to 7 (Very energetic) |
| **Academic Cognitive Performance** | During this episode, how focused were you on your academic task? | 1 (Very distracted) to 7 (Very focused) |
|  | How well were you performing your academic task? | 1 (Very poorly) to 7 (Very well) |
|  | How cognitively demanding was your academic activity? | 1 (Not at all demanding) to 7 (Extremely demanding) |
| **Emotional Regulation** | Did you intend to change your mood through music? | Yes, No, Unsure |
|  | To what extent did music help you achieve your mood goal? | 1 (Not at all) to 7 (Completely) |

**Table S2: Cronbach's Alpha Reliability Coefficients for All Measures**

| **Measure** | **Number of Items** | **Cronbach's α** |
| --- | --- | --- |
| Gold-MSI Total Score | 39 | .92 |
| Gold-MSI Active Engagement | 9 | .88 |
| Gold-MSI Perceptual Abilities | 9 | .85 |
| Gold-MSI Musical Training | 7 | .90 |
| Gold-MSI Emotions | 6 | .84 |
| Gold-MSI Musical Skills | 8 | .87 |
| TAS-20 Total Score | 20 | .86 |
| TAS-20 Difficulty Identifying Feelings | 7 | .83 |
| TAS-20 Difficulty Describing Feelings | 5 | .79 |
| TAS-20 Externally Oriented Thinking | 8 | .74 |
| TIPI Openness | 2 | .71 |
| TIPI Conscientiousness | 2 | .68 |
| TIPI Extraversion | 2 | .72 |
| TIPI Agreeableness | 2 | .70 |
| TIPI Neuroticism | 2 | .73 |
| MMR Entertainment | 3 | .76 |
| MMR Diversion | 3 | .78 |
| MMR Discharge | 3 | .74 |
| MMR Solace | 3 | .79 |
| ESM Pre-Listening Mood (Valence) | Single item | - |
| ESM Pre-Listening Mood (Arousal) | Single item | - |
| ESM Post-Listening Mood (Valence) | Single item | - |
| ESM Post-Listening Mood (Arousal) | Single item | - |
| ESM Academic Focus | Single item | - |
| ESM Academic Performance | Single item | - |
| ESM Academic Cognitive Demand | Single item | - |

*Note: Gold-MSI = Goldsmiths Musical Sophistication Index; TAS-20 = Toronto Alexithymia Scale; TIPI = Ten-Item Personality Inventory; MMR = Music in Mood Regulation Scale; ESM = Experience Sampling Method. Single-item measures do not have Cronbach's alpha coefficients.*

**SUPPLEMENTARY FIGURES**

**Supplementary Figure S1: Survey Flow Diagram**

Flow diagram illustrating the complete survey procedure for each listening episode. The diagram depicts the sequence of survey sections including pre-listening assessment, music listening period, and post-listening assessment with all measured variables.

**Figure S2: Comparison of Listening Purposes by Academic Context**

Grouped bar chart comparing listening purposes during academic versus non-academic activities. The y-axis represents the percentage of episodes for each listening purpose. Focus and concentration purposes were more common during academic activities, while mood regulation and background listening were more common during non-academic activities.
